# Anti‐Chi3L1 antibody suppresses lung tumor growth and metastasis through inhibition of M2 polarization

**DOI:** 10.1002/1878-0261.13152

**Published:** 2021-12-20

**Authors:** Ji Eun Yu, In Jun Yeo, Dong Ju Son, Jaesuk Yun, Sang‐Bae Han, Jin Tae Hong

**Affiliations:** ^1^ College of Pharmacy and Medical Research Center Chungbuk National University Cheongju‐si Korea

**Keywords:** anticancer therapy, chitinase 3‐like 1, humanized antibody, plasminogen, STAT6

## Abstract

Chitinase 3‐like 1 (Chi3L1) is associated with various biological processes, such as inflammation, tissue repair, proliferation, cell survival, invasion, and extracellular matrix remodeling. Recent studies indicated that Chi3L1 is critical for cancer development and metastasis. In this study, we demonstrate that Chi3L1 serum and tissue levels were significantly increased in lung cancer patients compared with controls. We previously developed an anti‐Chi3L1‐humanized antibody, and here, we investigate its antitumor and antimetastatic effect. The anti‐Chi3L1 antibody attenuated tumor growth and metastasis both *in vitro* and *in vivo* in a lung cancer mouse model. These inhibitory effects are associated with signal transducer and activator of transcription 6 (STAT6)‐dependent M2 polarization inhibition. Proteomics analysis revealed that plasminogen (PLG) interacts with Chi3L1 and affects M2 polarization. Chi3L1 plays a critical role in lung cancer progression, and the anti‐Chi3L1 antibody could be a new anticancer therapy.

## Graphical abstract

High Chi3L1 expression was correlated with poor overall survival in patients with lung cancer (LC). Here, we show that humanized anti‐Chi3L1 antibody attenuated tumor growth and metastasis *in vivo* via STAT6‐dependent PLG signaling and M2 polarization inhibition. Proteomics analysis revealed that plasminogen interacted with Chi3L1 and affected M2 polarization. Hence, inhibition of Chi3L1 could be considered as a novel therapeutic strategy for patients with LC.

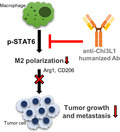

**Abbreviations**
AUC: area under the curve
CEACAM: carcinoembryonic antigen
Chi3L1: chitinase 3‐like 1
CM: conditioned medium
IL‐13: interleukin 13
IL‐4: interleukin 4
IPA: ingenuity pathways analysis
LLC: Lewis lung cancer
nano‐LC: nano‐high‐performance liquid chromatography
NSCLC: non‐small‐cell lung cancer
PD‐L1: programmed cell death 1 ligand 1
PLA: plasmin
PLG: plasminogen
PMA: phorbol 12‐myristate 13‐acetate
ROC: receiver operating characteristic
SCLC: small‐cell lung cancer
STAT6: signal transducer and activator of transcription 6
VEGF: vascular endothelial growth factor

## 1 Introduction

Chitinase 3‐like 1 (Chi3L1, YKL‐40 and human cartilage gp‐39), a chitinase‐like protein, is evolutionarily well conserved in mammalians [1]. In mammals, Chi3L1 maintains homeostasis in many organs. Chi3L1 is expressed in monocytes, macrophages, neutrophils, cultured chondrocytes and synovial cells and is involved in cell proliferation and survival. It has a mitogenic effect on skin, lung, fibroblast and synoviocytes cells [1, 2, 3, 4, 5, 6, 7]. Chi3L1 is present in a healthy human body but increases in various diseases, including rheumatoid arthritis, osteoarthritis, and cancers [5, 8, 9, 10]. Chi3L1 is expressed in many cancer cells and promotes cancer cell proliferation, macrophage recruitment, and blood vascular formation [5, 9, 10, 11]. A high level of Chi3L1 is correlated with poor prognosis in various human carcinomas such as leukemia, lymphoma, breast cancer, and lung cancer [5, 8, 11, 12]. Chi3L1 could be an independent prognostic markers for metastatic cancer. We previously found that Chi3L1 could be a significant new therapeutic cancer target [13].

According to a statistical analysis by the National Institutes of Health National Cancer Institute, there were 228 820 lung cancer patients in 2020, the second highest incidence among all cancers and 135 720 people died of lung cancer, the highest death rate among all cancers [14, 15]. In the past five years, the overall cancer survival rate has been 84.6%, while in lung cancer the survival rate has been 20.5%. Patients with lung cancer have a poor prognosis compared with other cancers [14]. It is difficult to diagnose lung cancer early because as there are often no symptoms. Lung cancer is divided into small‐cell lung cancer (SCLC) and non‐small‐cell lung cancer (NSCLC) according to pathological organizational standards such as the size and shape of the cancer cells. Recent cancer research shows the effects of therapy while minimizing side effects.

Monoclonal antibodies and biological inhibitors have recently emerged as effective therapies. Although monoclonal antibodies are potentially therapeutic agents, these nonhuman antibodies are considered foreign antigens in the human body, which causes immune responses and a short half‐life. Their therapeutic effects are therefore limited [16, 17, 18]. Repeated monoclonal antibody injections cause an immune response, making them difficult to use as a continuous treatment [18, 19]. To address these problems, a technique has been developed to replace antibodies with humanized monoclonal antibodies. Approximately 50 humanized antibodies have been clinically approved by the U.S. FDA, including humanized IgG1 for VEGF (bevacizumab; Avastin) and humanized IgG1 for HER2 (trastuzumab) [20, 21, 22]. VEGF and EGFR are well‐known tumorigenesis factors that have been studied as targets for various cancer signaling pathways. Many agents that inhibit the signaling of these proteins are being developed, but they have side effects and affects normal cells [23, 24]. Novel targets are needed that can increase the response rate of existing treatments and reduce side effects. Our previous study found Chi3L1 to be highly relevant for lung cancer compared with other targets including EGF [13]. Based on this study, we developed the anti‐Chi3L1‐humanized antibody using a Fab fragment [25]. In this study, we aim to identify the mechanism of action of this antibody as a lung cancer therapy.

## 2 Materials and methods

### 2.1 *In vivo* tumor growth and metastasis model

Eight‐week‐old male C57BL/6 mice were purchased from DBL (Eumsung, Korea). Animals were maintained under controlled conditions of temperature and light. They were provided standard mice feed and water *ad libitum*. For tumor growth model, Lewis lung cancer (LLC) cells [3 × 10^5^ cells/200 μL in phosphate‐buffered saline (PBS) with a 27‐gauge needle] were injected subcutaneously to 8‐week‐old C57BL/6 mice. Next day, 0.5 mg per kg of anti‐Chi3L1 antibody or Avastin was injected into intravenously twice a week for 4 weeks. The anti‐Chi3L1 antibody was isolated and purified in our previous study [25]. The Avastin was purchased from Roche (Basel, Switzerland).

For metastasis model, A549 cells (1 × 10^7^ cells/200 μL in PBS with a 27‐gauge needle) were injected intravenously to 8‐week‐old C57BL/6 mice and 0.5 mg per kg of anti‐Chi3L antibody or vehicle was injected into intravenously twice a week for 8 weeks. B16‐F10 mouse melanomas were injected subcutaneously (2 × 10^5^ cells/200 μL in PBS with a 27‐gauge needle) to 8‐week‐old C57BL/6 mice. Next day, 0.5 mg per kg of anti‐Chi3L1 antibody was inoculated through intravenous injection and these injections were performed every 3 days for 3 weeks. Since 15 days after melanoma injection, the tumor volume of the animals was monitored every 5 days.

The tumor volumes were measured with Vernier calipers and calculated by the following formula: (*A* × *B* ^2^)/2, where *A* is the larger and *B* is the smaller of the two dimensions. At the end of the experiment, the animals were sacrificed with inhalants using carbon dioxide. The tumors were separated from the surrounding muscles and dermis, and excised. Animal experiments were performed modifying the method described previously [26].

All protocols involving mice in this study were reviewed and approved by the Chungbuk National University Institutional Animal Care and Use Committee (IACUC) and complied with the Korean National Institute of Health Guide for the Care and Use of Laboratory Animals (CBNUA‐1073‐17‐01).

### 2.2 Hematoxylin and eosin (H&E) staining and Immunohistochemistry (IHC)

Mice tissues were dissected and immediately fixed in 4% formaldehyde solution, followed by dehydration in a graded ethanol series (70–100%) and embedding in paraffin. The tissues were then sectioned (8 μm thick) with a microtome (Sakura Finetek Europe BV, Torrance, CA, USA) and stained with hematoxylin and eosin. Sections were viewed under a light microscope (Olympus, Tokyo, Japan). For IHC, the tumor and lung tissue sections were blocked with 3% normal goat serum diluted in PBS, for 30 min; the sections were then incubated with antibodies for Chi3L1, MMP9, cyclin D1, CD86, CD206, plasminogen, and pSTAT6, at the appropriate dilution in blocking serum, for overnight at 4 °C. The slides were washed in PBS, followed by the avidin–biotin–peroxidase complex (#PK‐6101, Vector Laboratories, Burlingame, CA, USA). The slides were washed, and the peroxidase reaction was developed with diaminobenzidine and peroxide (#SK‐4100, Vector Laboratories), mounted in Aqua‐Mount, and evaluated under a light microscope (Olympus).

### 2.3 Cell culture and collection of conditioned medium (CM)

A549, THP‐1, Raw 264.7, and LLC cells were obtained from the American Type Culture Collection (Manassas, VA). A549 cells were cultured in RPMI 1640 medium supplemented with 10% heat‐inactivated fetal bovine serum (FBS), 100 μg·mL^−1^ penicillin, and 100 μg·mL^−1^ streptomycin. Human monocytic THP‐1 macrophages were cultured in RPMI supplemented with 10% heat‐inactivated FBS, 100 ng·mL^−1^ penicillin, 100 μg·mL^−1^ streptomycin, and 50 μm ß‐mercaptoethanol. Raw 264.7 and LLC cells were cultured in DMEM supplemented with 10% heat‐inactivated FBS, 100 μg·mL^−1^ penicillin, and 100 μg·mL^−1^ streptomycin. Cell cultures were maintained in an incubator with a humidified atmosphere of 5% CO_2_ at 37 °C. Cell culture was performed as described previously [13]. THP‐1 monocytes are differentiated into macrophages by 24‐h incubation with 100 μg·mL^−1^ phorbol 12‐myristate 13‐acetate (PMA; #P8139, Sigma‐Aldrich, St Louis, MO, USA) followed by 24‐h incubation in RPMI medium. M1 macrophages were differentiated by using recombinant LPS 10 pg·mL^−1^ (#L2630, Sigma‐Aldrich). M2 macrophages were differentiated by using 10 ng·mL^−1^ recombinant human interleukin 4 (IL‐4; #204‐IL, R&D System Inc., Minneapolis, MN, USA) and human interleukin 13 (IL‐13; #213‐ILB, R&D system Inc.). A549 cells were cultured in RPMI with 10% FBS. Then, the supernatants were discard and changed into serum‐free medium for further 24 h, after which the new supernatants of these cells were obtained as conditioned medium (CM).

### 2.4 Cell lysate preparation and western blotting

Lung tumor and metastatic lung tissues were homogenized with lysis buffer [50 mm Tris–Hcl (pH 7.6), 0.1% Triton X‐100, 0.25 mm NaCl, 2 mm EDTA with protease inhibitor and protease inhibitor] and lysed by 60‐min incubation on ice. The tissue and cell lysate were centrifuged at 13 000 ***g*** for 20 min at 4 °C. THP‐1 macrophage cells were washed with ice‐cold PBS and lysed on ice using lysis buffer supplemented with complete protease inhibitor. Total 10 μg of proteins was subjected to SDS/PAGE for separation and then transferred to PVDF membrane. PVDF membrane was incubated with specific primary antibodies, and following HRP‐conjugated secondary antibodies incubation, desired proteins were detected using ECL substrate (#WBKLS0500, Millipore, Billerica, MA, USA) and visualized by using a FUSION Solo S chemiluminescence detection system (Vilber Lourmat, Collégien, France). The primary antibodies were as follows: anti‐Chi3L1 (dilution 1 : 1000; #ab77528, Abcam, Cambridge, UK), anti‐IL13Rα2 (dilution 1 : 1000; A2043, Abclonal, Wuhan, China), anti‐Cdk2 (dilution 1 : 1000; #05‐596, Sigma‐Aldrich), anti‐Cdk4 (dilution 1 : 1000, #A0366, Abclonal), anti‐Cdk6 (dilution 1 : 1000; #sc‐7961, Santa Cruz Biotechnology, Dallas, TX, USA), anti‐cyclin D1 (dilution 1 : 1000; #ab134175, Abcam), anti‐cyclin E1 (dilution 1 : 1000, #A14225, Abclonal), anti‐MMP2 (dilution 1 : 1000; ab97779, Abcam), anti‐MMP9 (dilution 1 : 1000; #ab38898, Abcam), anti‐MMP13 (dilution 1 : 1000; #ab39012, Abcam), anti‐PCNA (dulutin 1 : 1000; #ab92552, Abcam), ant‐CD86 (dilution 1 : 1000, #91882, Cell Signaling, Boston, MA, USA), anti‐iNOS (dilution 1 : 1000, #PA1‐036, Invitrogen, Carlsbad, CA, USA), anti‐CD206 (dilution 1 : 1000; #ab64693, Abcam), anti‐ARG1 (dilution 1 : 1000; #93668, Cell Signaling Technology), anti‐Cox2 (dilution 1 : 1000; #ab15191, Abcam), antiplasminogen (dilution 1 : 1000; #Ab154560, Abcam), anti‐STAT6 (dilution 1 : 500; #ab28829, Abcam and dilution 1 : 1000; #54554, Cell Signaling), antiphosphorylated STAT6 (dilution 1 : 1000; #sc‐374021), anti‐Lamin B1, anti‐α‐tubulin (dilution 1 : 1000, #sc‐5286, Santz Cruz), anti‐Myc (dilution 1 : 2000, #AE070, Abclonal), and anti‐β‐actin (dilution 1 : 5000, sc‐517582, Santa Cruz).

### 2.5 RT‐qPCR assay

Total RNA was isolated from tissue and cell samples using the RiboEx Total RNA (#301‐001, GeneAll Biotechnology Co., Seoul, Korea) and reverse‐transcribed into CDNA using High‐Capacity cDNA Reverse Transcription kit (#4368813, Applied Biosystems, Foster City, CA, USA) according to manufacturer's protocol. Real‐time PCR was performed using a SYBR Green PCR Master mix (#4344463, Applied Biosystems) and analyzed using an ABI PRISM 7700 Sequence Detection system (Applied Biosystems). All primers used for RT‐qPCR were designed using ‘Primer3’ on website and purchased from Bioneer Corp. (Daejeon, Korea).

### 2.6 Immunocytochemistry

THP‐1 was treated with 100 ng·mL^−1^ PMA for 48 h and incubated with A549 CM. 1 μg·mL^−1^ anti‐Chi3L1 antibody was incubated for 0.5 h before A549 CM induction. Cells were fixed with 4% paraformaldehyde in PBS for 15 min at room temperature. Cells then were permeabilized with 100% cold‐methanol for 5 min and blocked by 4% BSA in PBS with 0.1% Triton X‐100 for 1 h. Primary antibodies were incubated for overnight at 4 °C and Alexa Fluor 488 (#A32723, #A32731, Invitrogen) or Texas Red (#T‐862, #T‐2767, Invitrogen)‐conjugated secondary antibodies were incubated for 1 h at room temperature. Fixed cells were incubated with 1 μg·mL^−1^ of DAPI (#D9542, Sigma‐Aldrich) for 5 min at room temperature and then covered with Fluoromount‐G Mounting Medium (#0100‐01, Southern Biotech, Birmingham, AL, USA). Cells were visualized using Ziess AxioObserver (Carl Zeiss, Oberkochen, Germany) fluorescence microscope system. Digital images were analyzed using the imagej (NIH, Bethesda, MD) or zen 2.1 (Carl Zeiss) software.

### 2.7 Cell migration assay

For wound‐healing assay, A549 cells were seeded in SPLScar Block cell culture dish (#201935, SPL, Pocheon, Korea). Block is composed of 500‐um‐thick wall to artificially generate cell free gap. The culture medium was immediately replaced by CM from THP‐1‐stimulated A549 CM with/without anti‐Chi3L1 antibody, and the cells were incubated for 24 h. Migrated cell images were visualized using a light microscope (Olympus) and analyzed using imagej software (NIH).

For transwell assay, A549 cells were seeded on upper chamber inserts (8.0 μm pore transwell; Corning Inc., Corning, NY, USA) and CM from THP‐1‐stimulated A549 CM with/without anti‐Chi3L1 antibody was added to the lower chamber. After incubation for 18 h, the cells were fixed with 4% formaldehyde for 5 min, permeated with 100% methanol for 15 min, and stained with 0.1% crystal violet for 20 min. In the upper chamber, nonmigrated cells were removed with a cotton swab. The migrated cells were visualized using a light microscope (Olympus) and analyzed using imagej software (NIH).

### 2.8 Enzyme‐linked immunosorbent assays (ELISA)

Blood was collected from various mice using BD Microtainer^®^ blood collection tubes (BD Biosciences, East Rutherford, NJ, USA). The collected blood was centrifuged at 1000 ***g*** for 15 min and stored at −80 °C. Concentration of mouse Chi3L1 and human Chi3L1 in mouse serum, human serum, and culture supernatants were measured by ELISA kit (#DY2649, #DY2599, R&D System Inc.). Concentration of mouse plasminogen and human plasminogen in human serum and culture supernatants were measured by ELISA kit (#MBS7608094, #MBS700494, MyBioSource, SanDiego, CA, USA) according to manufacturer’s protocol. Concentration of human PD‐L1, human VEGF, and human CEACAM in human serum was measured by ELISA kit (#MBS2702472, #MBS355343, #MBS7240709, MyBioSource).

### 2.9 Cell lysis and protein digestion

Cell pellets were stored at −80 °C until cell lysis was performed. Lysis of cell pellets was done at room temperature. Biological replicates (one cell pellet from one cell line) were processed simultaneously to minimize the effect of error. Pellets were resuspended in 100 μL 8 m urea (Merck, Branchburg, NJ, USA) was added. Protein concentration was estimated with the bicinchoninic acid assay (Pierce, Rockford, IL, USA). Proteins were reduced with 10 mm dithiothreitol (Sigma) and alkylated 25 mm iodoacetamide (Sigma). Samples were diluted in 50 mm AmBic (Promega, Madison, WI, USA) and trypsinized overnight at 37 °C at a trypsin/protein ratio of 1 : 50, w/w. The resulting peptide mixture was lyophilized overnight and digested peptides were cleaned by flowing through a Oasis HLB 1cc (10 mg) solid‐phase extraction (SPE) catridges (Oasis, Milford, MA, USA). Samples were dried using a Speed‐Vac (Thermo Savant, Holbrook, NY, USA) and stored at −80 °C until time for analysis. The peptides digestion protocols were performed modifying the method described previously [27].

### 2.10 LC‐MS/MS for global proteomics

LC‐MS/MS was performed as described previously [28]. Nano‐high‐performance liquid chromatography (nano‐LC) analysis was performed using an Easy n‐LC 1000 system (Thermo Fisher Scientific, San Jose, CA, USA). The column (15 cm × 75 μm) was packed in‐house with Jupiter 2 μm, 100 Å pore size C18 beads (Phenomenex, Torrance, CA, USA). Mobile‐phase A for LC separation consisted of 0.1% formic acid in deionized water and the mobile‐phase B consisted of 0.1% formic acid in acetonitrile. For analysis of fractionated samples, the mobile phase was programmed from 5% B over 10 min, 5% B to 30% B over 35 min, 30% B to 90% B over 12 min, and finally to 90% B to 5% B over 13 min at a flow rate of 300 nL·min^−1^. An Q‐ExactiveTM mass spectrometer (Thermo Fisher) was used for MS analyses and was operated with Xcalibur (version 2.1, Thermo Fisher) to generate peak lists. For peptide ionization, 2400 V was applied and a 250 °C capillary temperature was used. The full‐scan event was collected using a *m*/*z* 350–2000 mass selection, an Q‐Exactive MS resolution of 70 000, a target automatic gain control (AGC) value of 1 × 10^6^, and a maximum injection time of 80 ms. Fragmentation was performed with a normalized collision energy of 25.

### 2.11 Pathway analysis

Proteins were functionally classified using a gene ontology system by biological processes, molecular activities, and cellular components using the protein discoverer 2.2 program (Thermo) and normalized protein abundance values were calculated. Fold changes of the proteins were calculated by comparing resistance cell line conditions to control. 12. The ingenuity pathways analysis (IPA; Ingenuity Systems, Redwood City, CA, USA) program generates proteomic data containing UniProt identifiers and fold changes in total identified proteins. Protein interactions, pathways, and functional networks were generated using IPA. Based on experimentally observed data, key analyzes were performed with direct or indirect settings of the relationships between molecules, and data sources were considered in the human database of the ingenuity knowledge base. IPA predicted possible upstream regulators of proteins that appeared to be inhibited or activated in this study, depending on the statistical results of protein expression following the fold change, the *Z*‐score.

### 2.12 Human samples

Human tissue and serum samples from lung cancer patients and normal controls (20 samples, respectively) were obtained from Chungbuk National University Hospital Biobank, Keimyung University Dongsan Hospital Biobank and the Biobank of Ajou University Hospital, members of Korea Biobank Network. All studies using human samples were conducted in accordance with the Declaration of Helsinki and were approved by the Ethics Committee of Chungbuk National University Medical Center (IRB No. CBNU‐201910‐BR‐941‐01). The experiments were undertaken with the understanding and written consent of each subject.

### 2.13 Web‐based analysis

The genetic alteration data of Chi3L1 were analyzed by cBioPortal (https://cbioportal.org/). The data were extracted from 13 dataset of Non‐small‐cell Lung Cancer section (6122 samples). Overall survival in lung cancer for Chi3L1 expression was obtained from online Kaplan–Meier plotter (http://www.kmplot.com; 209396_s_at; Histology set as adenocarcinoma).

### 2.14 Statistical analysis

Statistical analyses were performed using the graphpad prism 5 software (GraphPad Software Inc., San Diego, CA, USA). All error bars reported are the standard deviation (SD) unless otherwise indicated. Pairwise comparisons were performed using Student’s *t*‐test. Multiple comparisons were using one‐way analysis of variance followed by Tukey’s tests. Differences between groups were considered significant at *P*‐values of < 0.05.

## 3 Results

### 3.1 Chi3L1 up‐regulation is associated with human lung cancer development

**Fig. 1:**
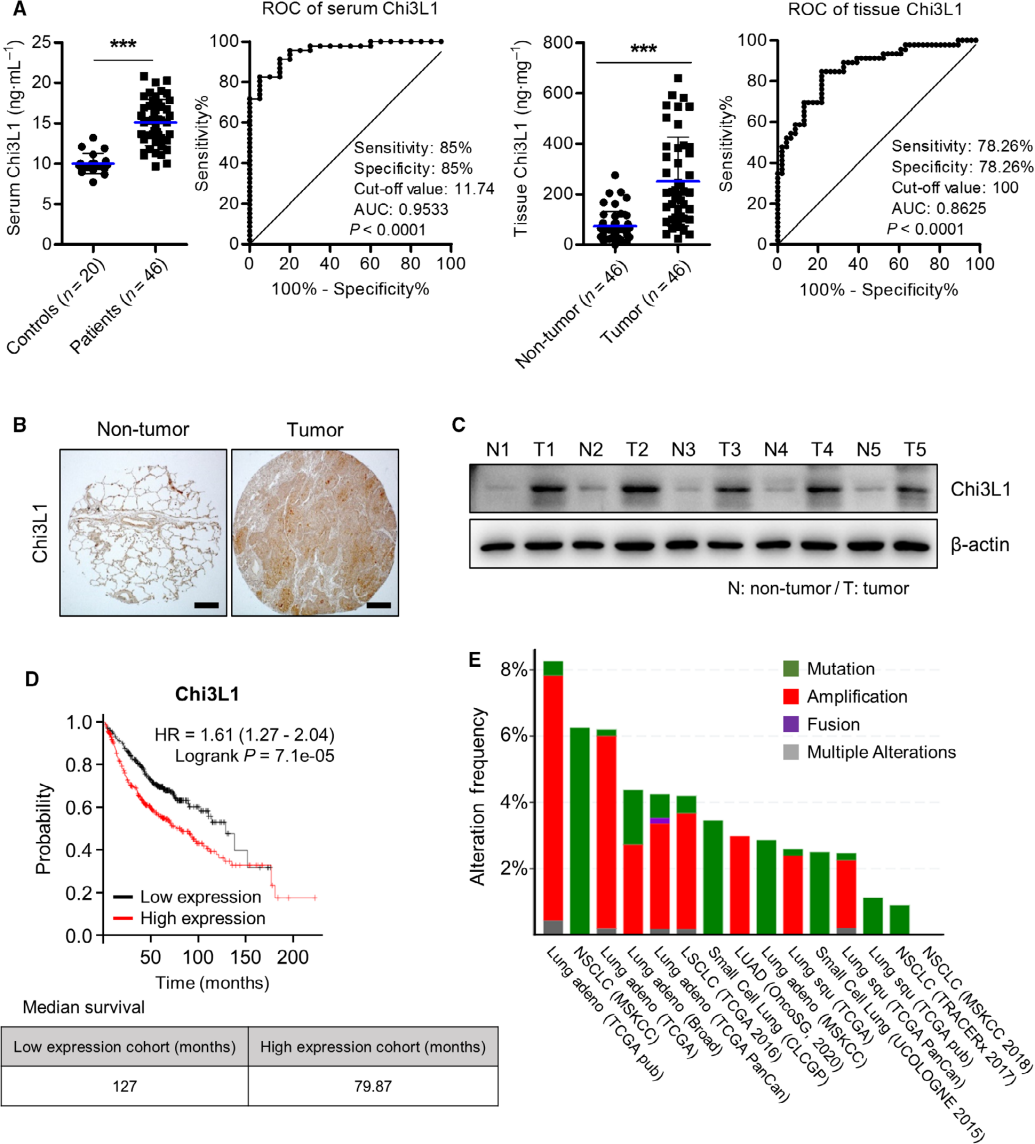
Chi3L1 plays an important role in human lung cancer. (A) Serum and tissue levels and receiver operating characteristic (ROC) curve of Chi3L1 in lung cancer patients and its controls. *n* = 20 for the control group; *n* = 46 for the patients group. ***P < 0.001 (unpaired two‐tailed *t*‐test). (B) Representative immunohistochemical images of lung cancer patients. Scale bar, 200 μm. Immunohistochemical staining were repeated from three independent experiments. (C) Chi3L1 expression in lung cancer patients was analyzed by Western blot. *n* = 5 per group. (D) The effects of Chi3L1 on the overall survival of lung cancer patients, using Kaplan–Meier plotter analysis. (E) Genetic alteration of Chi3L1 in lung cancer patients using cBioPortal.

Chitinase catalyzes the hydrolysis of chitin, a glycolytic molecule found in insect exoskeletons and fungi cell walls [29]. However, Chi3L1 is associated with inflammation, fibrosis, solid tumors, and asthma. Our previous studies indicated that Chi3L1 is critical for cancer development and metastasis [13, 26, 30, 31]. To verify the expression of Chi3L1 in lung cancer, we determined the protein level of Chi3L1 in human lung cancer patients and found that it was overexpressed in the serum and tissue of lung cancer patients compared with its controls (Fig. 1A–C). Chi3L1 was stage dependently increased in serum and tissue of human lung cancer patients (Fig. S1A,B). Receiver operating characteristic (ROC) curve analysis of serum Chi3L1 showed a sensitivity, specificity, cutoff value, and area under the curve (AUC) of 85%, 85%, 11.74 ng·mL^−1^, and 0.9533, respectively (Fig. 1A). ROC curve analysis of tissue Chi3L1 a showed sensitivity, specificity, cutoff value, and AUC of 78.26%, 78.26%, 100 ng·mg^−1^, and 0.8625, respectively (Fig. 1A). We compared other lung cancer biomarkers, vascular endothelial growth factor (VEGF), programmed cell death 1 ligand 1 (PD‐L1), and carcinoembryonic antigen (CEACAM), with Chi3L1 in the serum of lung cancer patients. Serum levels of VEGF, PD‐L1, and CEACAM were not different compared with healthy controls except the PD‐L1 level at stage 3 (Fig. S1C). We further investigated the relationship between overall patient survival and Chi3L1 levels using a Kaplan–Meier plotter. High levels of Chi3L1 expression were correlated with poor overall survival in lung cancer patients (lung adenocarcinoma) (Fig. 1D). We further analyzed genetic alterations of Chi3L1 in human lung cancer using cBioPortal. Amplification and mutation were the most common genetic events (Fig. 1E). These results suggest that Chi3L1 is a key regulator of human lung cancer development.

### 3.2 Anti‐Chi3L1 antibody inhibited lung cancer growth

**Fig. 2:**
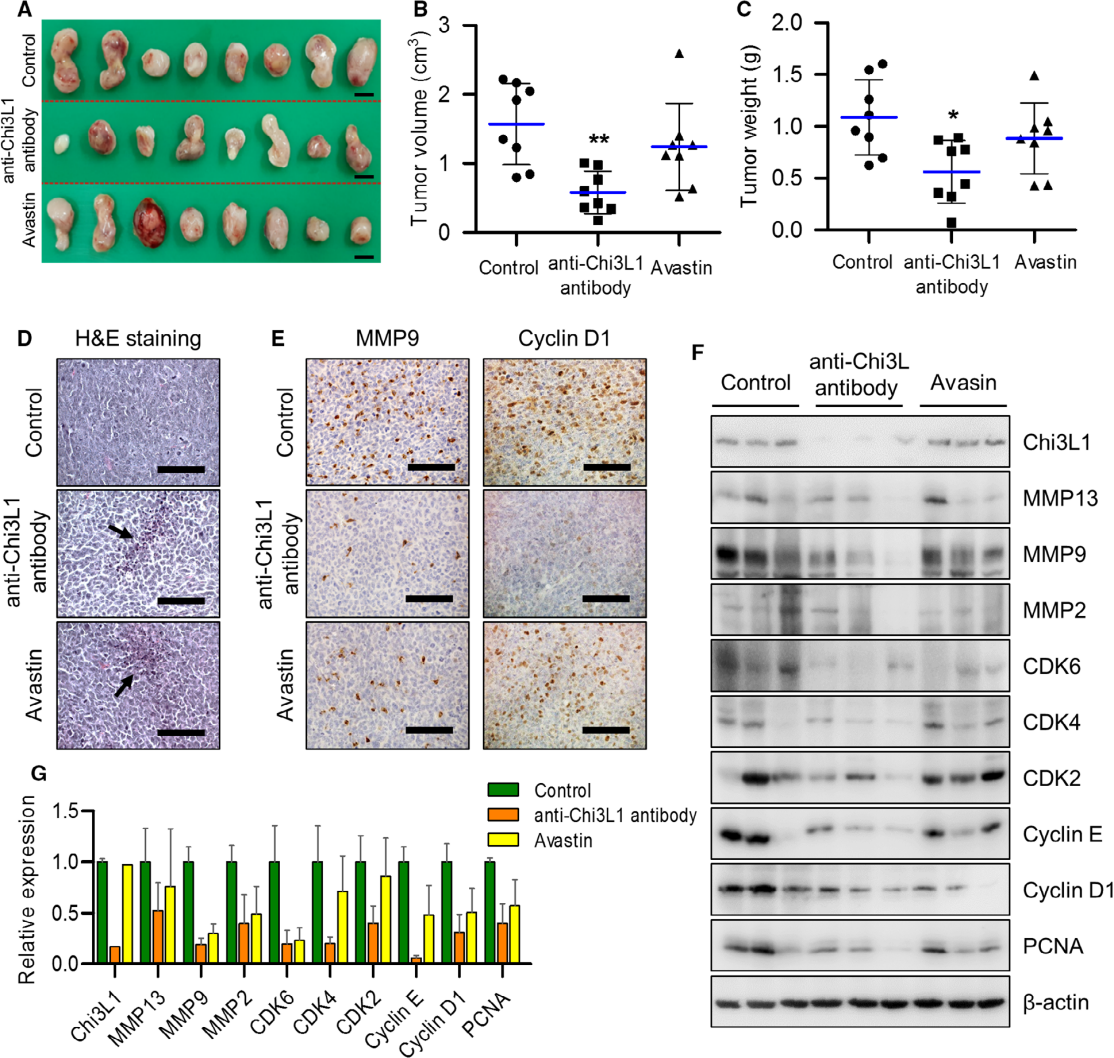
Anti‐Chi3L1 antibody blocks tumor growth *in vivo*. (A–C) Lewis lung cancer (LLC) cells (3 × 10^5^ cells) were injected subcutaneously to induce lung cancer tumors and 0.5 mg·kg^−1^ of vehicle, anti‐Chi3L1 antibody, or Avastin were intravenously administrered twice a week for 4 weeks. n = 8 per group. (A) Representative image of tumors obtained from mice in each group. Scale bar, 1.5 cm. (B, C) Tumor size and weight were measured, and tumor volume was calculated with the formula; *V* = *L* × *W*2 × 0.5 (*n* = 8). *P < 0.05; **P < 0.01; (one‐way ANOVA). (D) H&E staining images of tumor tissues were excised from each group. Arrows indicate apoptotic cells. H&E staining were repeated from three independent experiments. Scale bar, 400 μm. (E) Representative immunohistochemical images of tumor tissues using anti‐MMP9 and anticyclin D1 antibodies in each group. Scale bar, 100 μm. (F) The tumor tissue extracts were subjected to immunoblot analysis with indicated antibodies. (G) The intensity of each band in (F) was measured and the ratio of the amount of each protein to β‐actin was calculated. Data are presented as mean ± standard deviation (SD) from two independent experiments.

Our previous studies generated high affinity anti‐Chi3L1 humanized monoclonal antibodies in human synthetic Fab phage display libraries [25]. In this study, we analyzed how blocking Chi3L1 protein with anti‐Chi3L1 humanized antibody affects the progression of lung cancer in a xenograft mouse model. We evaluated the tumor growth inhibition of the anti‐Chi3L1 antibody using a cell line‐induced lung cancer model via subcutaneous injection of LLC cells. The day after subcutaneous injection of LLC cells, anti‐Chi3L1 antibody, Avastin, or vehicle were intravenously injected with 0.5 mg·kg^−1^ twice a week for 4 weeks. We used VEGF inhibitor Avastin, as a positive control among several antibodies that were effective inhibitors of lung cancer growth and metastasis. As shown in Fig. 2A,B, the volume of vehicle‐treated tumor tissues (1.57 ± 0.59) was dramatically decreased to 0.58 ± 0.31 in anti‐Chi3L1 antibody‐treated mice, and the volume of tumor tissue was lower compared with Avastin (Fig. 2A,B). Furthermore, the tumor occupied 1.08 ± 0.36 of the tissue weight in vehicle‐treated mice, which significantly decreased to 0.56 ± 0.30 with anti‐Chi3L1 antibody (Fig. 2C). Chi3L1 tissue and serum levels significantly decreased with anti‐Chi3L1 antibody administration (Fig. S2A,B). Chi3L1 immunohistochemistry decreased significantly in the tumor tissue of mice treated with anti‐Chi3L1 antibody (Fig. S2C). Hematoxylin and eosin (H&E) staining revealed that the control tumor tissues had large nuclei and tight spaces and were present in mitotic cells, a characteristics of cancer cells. However, in anti‐Chi3L1 antibody‐ or Avastin‐treated tumor tissues, the number of mitotic cells was lower than in vehicle‐treated tumor tissues, and the number of dead cells was higher (Fig. 2D). We found that the anti‐Chi3L1 antibody inhibited MMP9 and cyclin D1 expression compared with the vehicle‐treated tumor tissues (Fig. 2E). Immune blot results showed that MMP2, MMP9, and MMP13 (migration marker proteins) expression decreased in tumor tissues of anti‐Chi3L1 antibody‐treated mice. The expression level of cell cycle‐related proteins, such as cyclin D1, cyclin E, Cdk2, Cdk4, Cdk6, and PCNA was also decreased in anti‐Chi3L1 antibody‐treated lung metastasis tumor tissue (Fig. 2F,G). These results suggest that the anti‐Chi3L1 antibody interferes with LLC lung tumor growth, and its inhibitory effect was superior to that of Avastin.

### 3.3 Anti‐Chi3L1 antibody blocked lung cancer metastasis *in vivo*

**Fig. 3:**
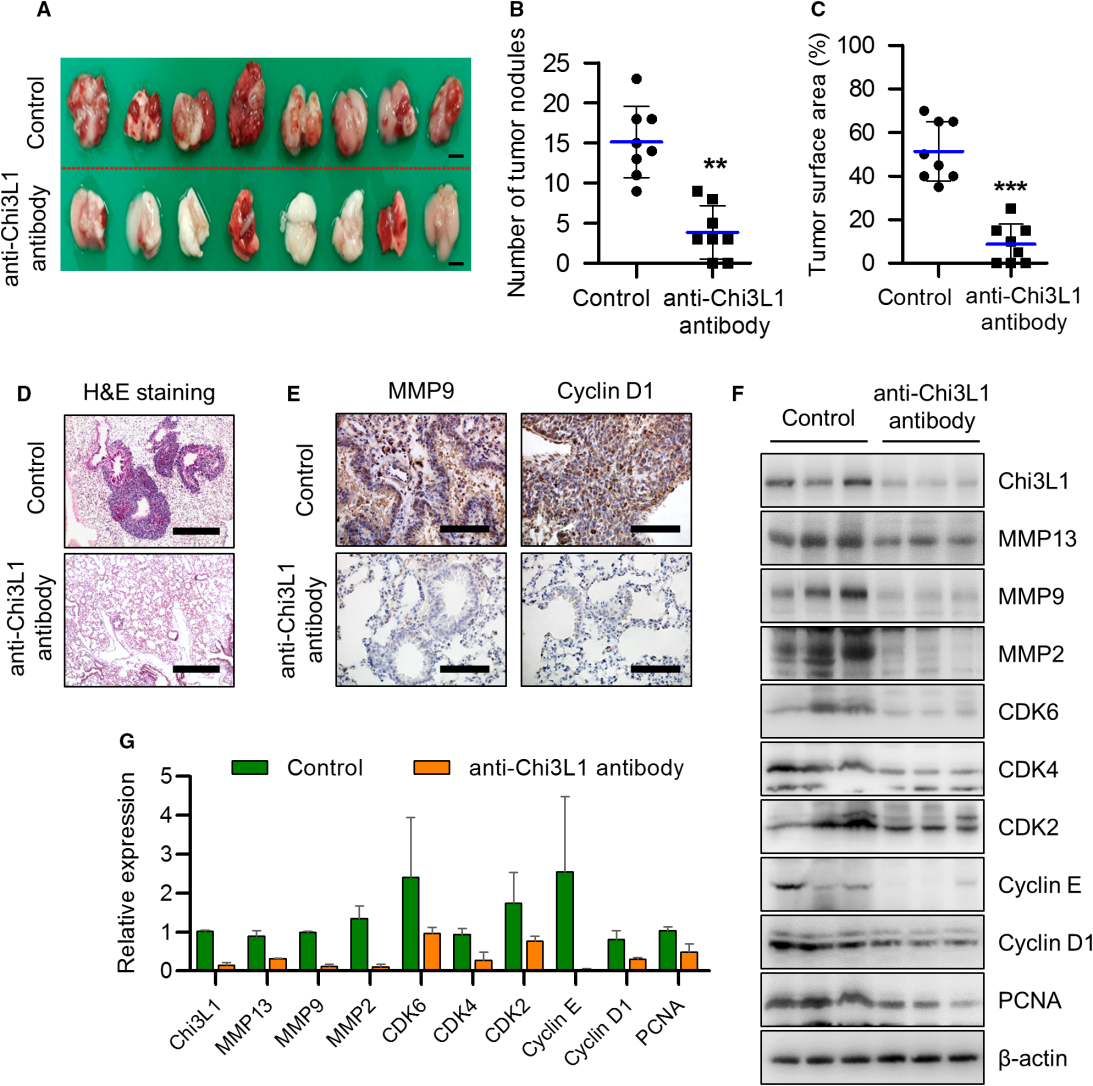
Anti‐Chi3L1 antibody blocks lung metastases *in vivo*. (A‐C) A549 cells (1 × 10^7^ cells) were injected intravenously anti‐Chi3L1 antibody or vehicle were injected intravenously twice a week for eight weeks. *n* = 8 per group. (A) Representative image of lung metastasis progression obtained from the mice in each group. Scale bar, 30 mm. (B) The number of metastatic nodules on the lung surface was counted and quantified (*n* = 8). **P < 0.01 (unpaired two‐tailed *t*‐test). (C) The tumor surface areas on the lung tissue were measured from the H&E staining images and quantified as a percentage of the total lung surface area (*n* = 8). ***P < 0.001; (unpaired two‐tailed *t*‐test). (D) H&E staining images of metastatic lung tissues excised from each group. H&E staining was repeated from three independent experiments. Scale bar, 200 μm. (E) Representative immunohistochemical images of lung tissues using anti‐MMP9 and anticyclin D1 antibodies in each group. Immunohistochemical staining was repeated from three independent experiments. Scale bar, 100 μm (F) The lung tissue extracts were subjected to immunoblot analysis with indicated antibodies. (G) The intensity of each band in (F) was measured and the ratio of the amount of each protein to β‐actin was calculated. Data are presented as mean ± standard deviation (SD) from two independent experiments.

To investigate whether the anti‐Chi3L1 antibody suppresses metastasis *in vivo*, A549 cells widely used as models for lung metastasis were injected into tail veins and the anti‐Chi3L1 antibody or vehicle were intravenously injected into the mice twice a week for eight weeks. The Chi3L1 protein level in anti‐Chi3L1 antibody‐treated lung metastasis tissue was slightly decreased, but the serum level was significantly reduced (Fig. S3A,B). The number of metastatic nodes in lung tissues was decreased in anti‐Chi3L1 antibody‐treated mice (Fig. 3A,B). The surface areas of metastatic lung were significantly reduced in anti‐Chi3L1 antibody‐treated mice compared with the controls (Fig. 3C). H&E staining analyses of lung tumors showed that vehicle‐injected mice presented well‐differentiated lung adenocarcinomas, but less lung metastasis was showed in anti‐Chi3L1 antibody‐treated mice (Fig. 3D). Immunohistochemistry analysis showed that the expression of Chi3L1 was inhibited in anti‐Chi3L1 antibody‐treated lung tumor tissues (Fig. S3C). The expression of MMP9 and cyclin D1 was also suppressed in anti‐Chi3L1 antibody‐treated lung metastasis tumor tissues (Fig. 3E). To confirm these results again, we analyzed the expression of metastasis and cell cycle‐related protein by immunoblot assay. As shown in Fig. 3F, anti‐Chi3L1 antibody significantly decreased the expression of cancer growth and migration‐associated proteins, such as PCNA, cyclin D1, cyclin E, Cdk2, Cdk4, Cdk5, MMP2 and MMP9 in A549 lung metastasis model tissues (Fig. 3F,G).

Although the B16‐F10 melanoma cells are not a lung cancer cell line, further investigation was conducted by selecting the B16‐F10 mouse melanoma cell line as a classic model that is highly likely to cause lung metastasis. B16‐F10 melanoma cells were injected into C57BL6/mice followed by intravenous anti‐Chi3L1 antibody at 0.5 mg·kg^−1^ twice a week for eight weeks. Anti‐Chi3L1 antibody‐treated mice had a greatly decreased tumor area and reduced number of tumor nodules (Fig. S4A–C). Histological analysis of metastatic lung adenomas showed that anti‐Chi3L1 antibody‐treated mice cells had a low‐density distribution, but the vehicle‐treated mice cells were densely distributed (Fig. S4D). Immunohistochemistry and western blotting analysis showed that the expression of Chi3L1, migration proteins, and cell cycle‐related proteins was significantly and consistently inhibited in those treated with anti‐Chi3L1 antibody (Fig. S4E–G). These results suggest that this anti‐Chi3L1 antibody is critical for the effective inhibition of lung metastasis.

### 3.4 Anti‐Chi3L1 antibody efficiently inhibits M2‐like macrophage polarization *in vivo*

**Fig. 4:**
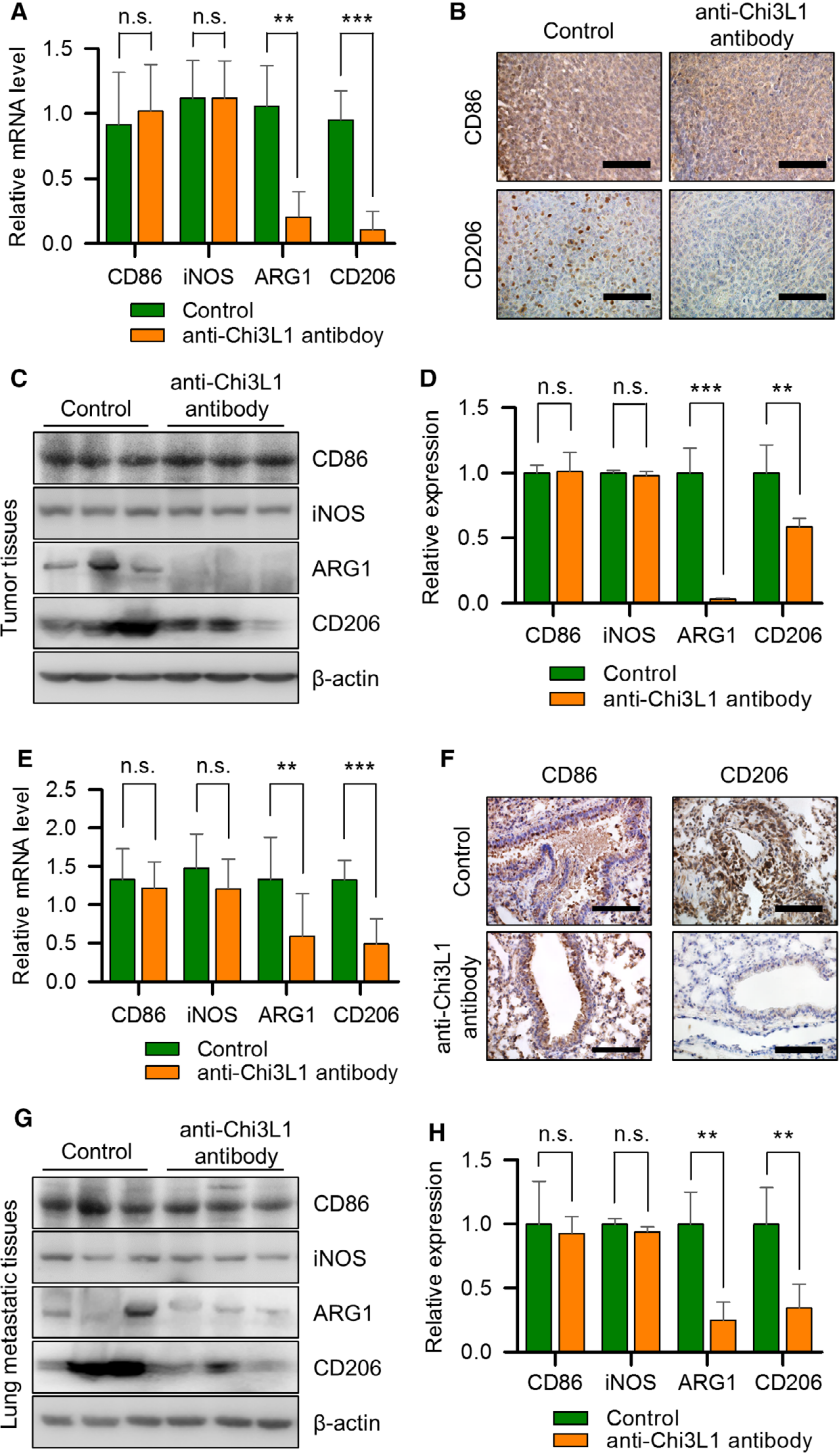
Anti‐Chi3L1 antibody inhibits M2‐like polarization in tumors and at metastatic site. (A–D) Lewis lung cancer (LLC) cells were injected subcutaneously to induce lung cancer tumors. Vehicle or anti‐Chi3L1 antibody was injected intravenously twice a week for 4 weeks. (A) Quantitative real‐time PCR analysis of M1‐ (CD86 and iNOS) and M2‐marker (CD206 and ARG1) gene mRNA expression levels in the tumor tissues of each group. Data are presented as mean ± standard deviation (SD) from three independent experiments. **P < 0.01; ***P < 0.001; n.s. (not statistically significant), *P* > 0.05 (unpaired two‐tailed *t*‐test). (B) Representative immunohistochemical images of tumor tissues using anti‐CD86 and anti‐CD206 antibodies. Immunohistochemical staining was repeated from three independent experiments. Scale bar, 100 μm. (C) The tumor tissue extracts were subjected to immunoblot analysis with M1‐ and M2‐marker proteins antibodies. (D) The intensity of each band in (C) was measured and the ratio of the amount of each protein to β‐actin was calculated. Data are presented as mean ± standard deviation (SD) from two independent experiments. **P < 0.01; ***P < 0.001; n.s. (not statistically significant), *P* > 0.05; (unpaired two‐tailed *t*‐test). (E‐H) A549 cells were injected intravenously into the mice. Anti‐Chi3L1 antibody was injected intravenously twice a week for eight weeks. (E) The real‐time qPCR analysis of M1‐ and M2‐marker gene mRNA expression levels in lung tissues of each group. Data are presented as mean ± standard deviation (SD) from three independent experiments. **P < 0.01; ***P < 0.001; n.s. (not statistically significant), *P* > 0.05; (unpaired two‐tailed *t*‐test). (F) Representative immunohistochemical images of lung tissues using anti‐CD86 and anti‐CD206 antibodies. Immunohistochemical staining were repeated from three independent experiments. Scale bar, 100 μm. (G) The lung tissue lysates were subject to immunoblot analysis with M1‐ and M2‐marker proteins antibodies. (H) The intensity of each band in (G) was measured and the ratio of the amount of each protein to β‐actin was calculated. Data are presented as mean ± standard deviation (SD) from two independent experiments. **P < 0.01; n.s. (not statistically significant), *P* > 0.05; (unpaired two‐tailed *t*‐test).

In tumor microenvironments, macrophages serve as M1 polarizes to prevent tumors and M2 polarization [32, 33, 34]. Several papers have reported that Chi3L1 is associated with Th2 immunity and M2 macrophage polarization activation [35, 36, 37]. We investigated whether anti‐Chi3L1 antibodies altered macrophage polarization in tumor tissue. The mRNA expression of the M1 (iNOS and CD86) and M2 markers (ARG1 and CD206) in tumor tissue was analyzed by RT‐qPCR. The data showed that M1 marker iNOS and CD86 were not affected by the anti‐Chi3L1 antibody, but the mRNA expression of the M2 markers CD206 and ARG1 was significantly decreased in the anti‐Chi3L1 antibody‐treated groups (Fig. 4A). Immunohistochemical staining of CD86 showed no significant difference between the two groups. Immunohistochemical staining of CD206 was decreased in the anti‐Chi3L1 antibody‐treated group (Fig. 4B). A Western blot was performed to explore changes to M1‐ and M2‐related proteins. Anti‐Chi3L1 antibody‐treated tumor tissues promoted ARG1 and CD206 expression inhibition compared with vehicle‐treated tumor tissues but did not change iNOS and CD86 expression (Fig. 4C,D). F4/80 and CD68 is well known as a macrophage marker proteins. Compared with the vehicle, the expression of CD68 and F4/80 was significantly reduced in anti‐Chi3L1 antibody‐treated tumor tissues (Fig. S5A–C). We assume that the anti‐Chi3L1 antibody may inhibit tumor growth by acting on macrophages.

To evaluate the impact of the anti‐Chi3L1 antibody on the polarization of macrophages in metastatic lung tissues, we detected the polarization of M1‐ and M2 markers in lung metastatic mice. Quantitative real‐time PCR was performed to measure the mRNA levels of M1‐ and M2‐like macrophages. CD206 and ARG1 mRNA expression was greatly reduced in anti‐Chi3L1 antibody‐treated lung metastatic tissues compared with the vehicle‐treated group (Fig. 4E). Immunohistochemistry analysis showed that, compared with untreated controls, the percentage of CD206^+^ macrophages was markedly decreased in the anti‐Chi3L1 antibody‐treated lung metastatic tissues (Fig. 4F). We investigated the anti‐Chi3L1 antibody‐mediated effect on the altered expression of macrophage polarization proteins in lung metastatic tumor tissues. The anti‐Chi3L1 antibody did not reduce iNOS and CD86 expression markedly. However, CD206 and ARG1 were dramatically reduced by the anti‐Chi3L1 antibody in lung metastatic tumor tissues (Fig. 4G,H). This suggests that the anti‐Chi3L1 antibody antagonizes the tumorigenesis induced by M2‐like polarized macrophages.

### 3.5 Anti‐Chi3L1 antibody abrogates M2‐like macrophage promoted lung cancer cell invasion and migration *in vitro*

**Fig. 5:**
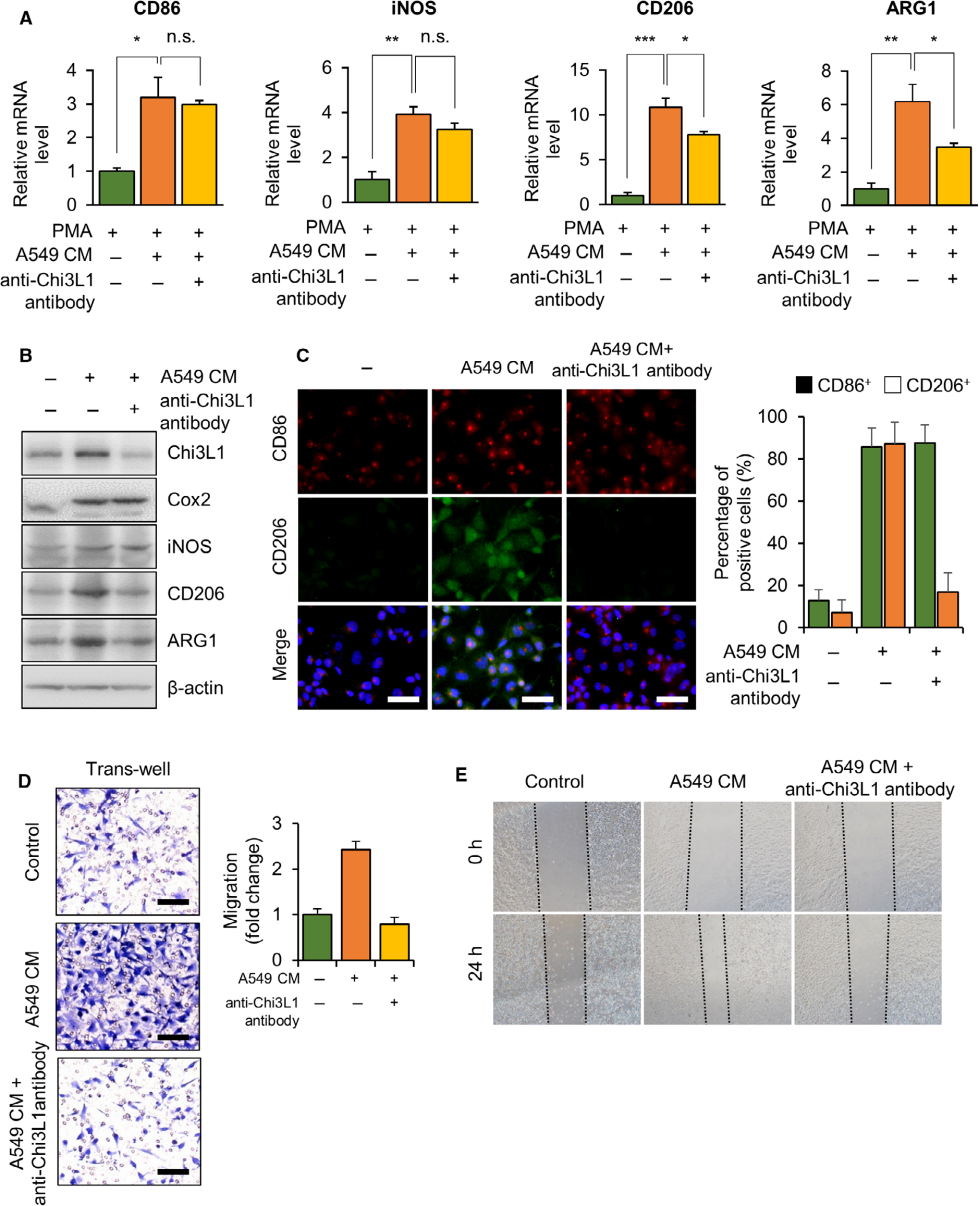
Anti‐Chi3L1 antibody inhibits M2‐like macrophage induced by A549 conditioned medium (CM) stimulation. (A‐C) THP‐1 was treated with phorbol 12‐myristate 13‐acetate (PMA) (100 ng·mL^−1^) and stimulated with A549 conditioned medium (CM) with or without anti‐Chi3L1 antibody (1 μg·mL^−1^). (A) RT‐qPCR analysis of M1‐ and M2‐marker gene mRNA levels. Data are presented as mean ± standard deviation (SD) from three independent experiments. *P < 0.05; **P < 0.01; ***P < 0.001; n.s. (not statistically significant), *P* > 0.05; (one‐way ANOVA). (B) The cell lysates were subject to immunoblot analysis with M1‐ and M2‐marker protein antibodies. (C) The 4% paraformaldehyde fixed cells were immunofluorescence stained with anti‐CD86 and anti‐CD206 antibodies. The percentage of CD86^+^ and CD206^+^ cells was calculated. Data are presented as mean ± standard deviation (SD) from three independent experiments. Scale bar, 50 μm. (D, E) THP‐1 was treated with phorbol 12‐myristate 13‐acetate (PMA) (100 ng·mL^−1^) and stimulated with A549 CM with or without anti‐Chi3L1 antibody (1 μg·mL^−1^) for 24 h. The culture medium was replaced with fresh media, and the supernatant medium was collected as macrophage‐conditioned medium(CM). (D) The effect of macrophage‐CM on A549 cell migration was evaluated by a Transwell assay. The A549 cells were seeded in the upper chamber and macrophage‐conditioned medium(CM) was placed into the lower chambers and incubated at 37 °C for 18 h. The migrated cells on the bottom chamber were stained with 0.1% crystal violet. Data are presented as mean ± standard deviation (SD) from three independent experiments. Scale bar, 100 μm. (E) Representative wound‐healing assay results of A549 cells treated macrophage‐conditioned medium (CM). For the wound‐healing assay, A549 cells were seeded in a plate; then, the cells were wounded with a straight scratch using a pipette tip and treated with macrophage‐conditioned medium (CM) for 18 h. Wound‐healing assay was repeated from three independent experiments.

To confirm our *in vivo* results, we analyzed whether the anti‐Chi3L1 antibody had an inhibitory effect on M2‐like polarization in THP‐1 macrophages. The A549 conditioned medium (CM) obtained from lung cancer cells was used to mimic the tumor microenvironment. To confirm the polarization of THP‐1 cells, the transcription level of M1 and M2 genes was studied by quantitative real‐time PCR. As shown in Fig. 5A, A549 CM triggered up‐regulation of M1‐ and M2‐like polarization marker genes. However, the anti‐Chi3L1 antibody did not cause remarkable inhibition of M1‐like polarization, whereas the up‐regulated mRNA expression of CD206 and ARG1 was diminished by this antibody. Significant up‐regulation of ARG1 and CD206 was observed when THP‐1 was stimulated with A549 CM, which was inhibited by the anti‐Chi3L1 antibody (Fig. 5A). Western blot analysis also demonstrated that the expression of iNOS, CD86, CD206, and ARG1 proteins was increased in the THP‐1 after A549 CM stimulation. In anti‐Chi3L1 antibody‐treated A549 CM stimulation, the protein levels of CD206 and ARG1, but not iNOS and CD86, significantly decreased compared with A549 CM stimulation (Fig. 5B). Furthermore, the M1‐ and M2‐polarized THP‐1 macrophages were stimulated by LPS and IL‐4/IL‐13, respectively. The simulation by LPS increased the protein and mRNA expression of CD86 and iNOS, which were not affected by anti‐Chi3L1 antibody treatment (Fig. S6A,B). Anti‐Chi3L1 antibody treatment affected IL‐4/IL‐13‐stimulated M2 polarization and greatly reduced CD206 and ARG1 protein and mRNA expression (Fig. S6C,D). Immunofluorescence staining showed that CD86^+^ and CD206^+^ fluorescence signals increased in A548 CM stimulation. In anti‐Chi3L1 antibody‐treated cells, there was no significant change in CD86^+^ fluorescence signals, and CD206^+^ fluorescence signals decreased (Fig. 5C). These result suggest that anti‐Chi3L1 antibody treatment reduced M2‐like polarization macrophages but does not affect M1‐polarized macrophages.

We also confirmed anti‐Chi3L1 antibody effects using murine macrophage RAW 264.7, which is commonly used to study macrophage polarization. The mRNA expression of CD86 and iNOS were significantly increased by the LPS stimulation, and there was no significant change by anti‐Chi3L1 antibody treatment (Fig. S7A). M2 polarization (CD206 and ARG1) increased by IL‐13/IL‐4 stimulation were significantly reduced by anti‐Chi3L1 antibody treatment (Fig. S7B). Fluorescence microscopy analysis result showed that IL‐13/IL‐4 stimulation increased the expression of ARG1 in RAW 264.7 macrophages, and the expression was reduced by anti‐Chi3L1 antibody treatment (Fig. S7C). Quantification of CD206 intensity revealed significant reduction in anti‐Chi3L1 antibody‐treated cells (Fig. S7C).

Anti‐Chi3L1 antibody treatment inhibited M2 polarization. Its effect on the migration of A549 cells was determined using a transwell assay. THP‐1 was incubated with A549 CM with and without anti‐Chi3L1 antibody. The medium was replaced with fresh medium. CM from THP‐1 treated with A549 CM promoted A549 cells invasions while CM from THP‐1 treated with both A549 CM and anti‐Chi3L1 antibody inhibited A549 cell invasions (Fig. 5D). Similarly, transwell assay in RAW 264.7 macrophages showed that the migration increased with IL‐13/IL‐4 treatment, and decreased cells invasion by anti‐Chi3L1 antibody treatment (Fig. S7D). We evaluated the migrating ability of A549 cells in various THP‐1 CMs with wound‐healing assay (Fig. 5E). We found that A549 CM‐stimulated macrophage‐CM significantly promoted cell migration, whereas a combination of A549 CM and anti‐Chi3L1 antibodies did not. These results suggest that anti‐Chi3L1 antibody could inhibit cell migration through M2‐like polarization inhibition.

### 3.6 STAT6 signaling pathway and anti‐Chi3L1 antibody‐mediated M2‐like polarization inhibition

**Fig. 6:**
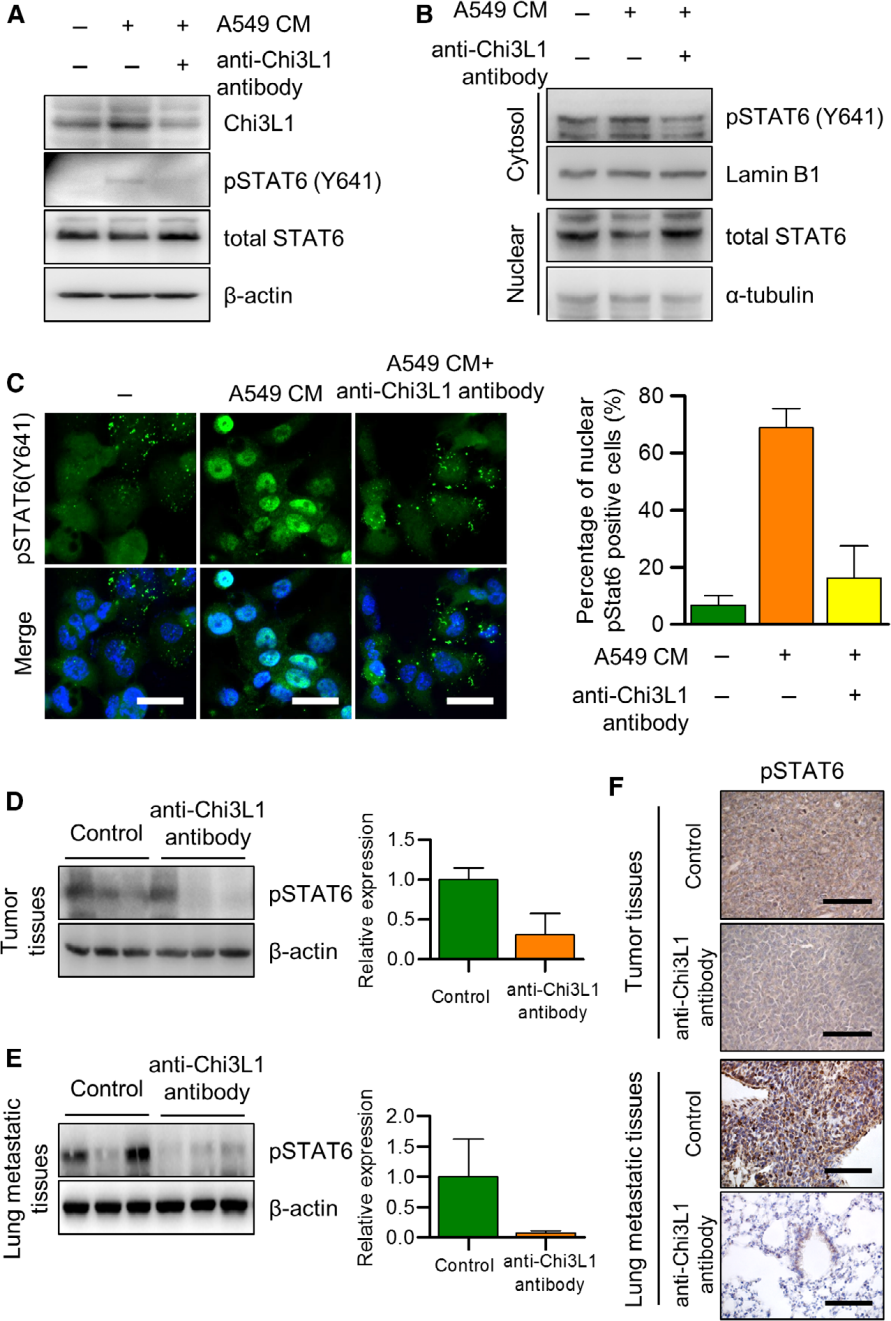
STAT6 is involved in the anti‐Chi3L1 antibody‐induced inhibition of M2‐like macrophage polarization. (A–C) THP‐1 was treated with phorbol 12‐myristate 13‐acetate (PMA) (100 ng·mL^−1^) and stimulated with A549 conditioned medium (CM) with or without anti‐Chi3L1 antibody (1 μg·mL^−1^). (A) Western blot was performed to measure the p‐STAT6. (B) Immunoblot of protein expression in cytoplasmic and nuclear fractions of THP‐1. Lamin B1 and α‐tubulin are the loading controls for nuclear and cytoplasmic fractions, respectively. (C) The fixed cells were immunofluorescence stained with p‐STAT6. The percentage of nuclear localized p‐STAT6 cells was calculated. Data are presented as mean ± standard deviation (SD) from three independent experiments. Scale bar, 20 μm. (D, E) The tumor and lung metastatic tissue samples were analyzed with immuno‐blotting with the p‐STAT6 antibody. Data are presented as mean ± standard deviation (SD) from three independent experiments. (F) The tumor and lung tissue samples were analyzed with immunohistochemistry with the p‐STAT6 antibody. Immunohistochemical staining were repeated from three independent experiments. Scale bar, 100 μm.

Given that STAT6 plays a key role in macrophage M2‐like polarization [38], we assessed whether it is involved in anti‐Chi3L1 antibody inhibited M2 polarization. Figure 6A indicates that the anti‐Chi3L1 antibody markedly reduced STAT6 phosphorylation, which was significantly increased in A549 CM‐stimulated THP‐1 macrophages (Fig. 6A). Also, STAT6 phosphorylation was reduced by anti‐Chi3L1 antibody in RAW 264.7 macrophages (Fig. S8A). Activation of STAT6 by M2 polarization includes phosphorylation of Try641 STAT6, followed by nuclear translocation, where it binds to specific DNA elements in the promoter region and activates gene transcription [39]. The fractions were validated by Western blot analysis using anti‐α‐tubulin in the cytosol fraction and anti‐Lamin B1 in the nuclear fraction. Phosphorylated Try641 STAT6 was decreased in the nuclear fraction of anti‐Chi3L1 antibody‐treated cells (Fig. 6B). Phosphorylated STAT6 translocation was confirmed by fluorescence microscopy, and phosphorylated STAT6 was almost translocated into the nucleus in A549 CM‐stimulated THP‐1 macrophages. More than 50% of anti‐Chi3L1 antibody‐treated cells inhibited phosphorylated STAT6 nuclear localization (Fig. 6C). Similar results were obtained in RAW 264.7 macrophages. We confirmed that phosphorylated STAT6 by IL‐13/IL‐4 stimulation was activated in the nucleus and localized to cytosol by anti‐Chi3L1 antibody. (Fig. S8B). These results indicate that anti‐Chi3L1 antibody reduced activation and nuclear localization of STAT6 in macrophages.

We confirmed that the anti‐Chi3L1 antibody inhibits STAT6 phosphorylation‐dependent M2 polarization. We examined whether STAT6 inhibitor AS1517499 and anti‐Chi3L1 antibody combination treatment showed additive effects on tumor migration by STAT6‐dependent M2 polarization. However, there was no additive effect, and our data indicated that anti‐Chi3L1 antibody treatment significantly reduced pSTAT6 expression (Fig. S9A). The combination treatment’s effect on A549 cell migrations was determined using a wound‐healing assay. The CM from THP‐1 stimulated A549 CM facilitated the migration of A549 cells, and single anti‐Chi3L1 antibody‐treated CM, single AS1517499‐treated CM, and anti‐Chi3L1 antibody combined with AS1517499‐treated CM showed similar inhibition of A549 cell migrations (Fig. S9B). A transwell assay showed no significant difference in cell invasion in a single anti‐Chi3L1 antibody treatment, single AS1517499 treatment, and combination anti‐Chi3L1 antibody and AS1517499 treatment (Fig. S9C).

To confirm the inhibition of STAT6 phosphorylation by the anti‐Chi3L1 antibody in a mouse tumor growth and metastasis model, an immunoblot assay and immunohistochemistry were performed using antiphosphorylated Try641 STAT6 antibodies. The results in Fig. 6D indicate that STAT6 phosphorylation was significantly reduced in tumor tissue in mice receiving the anti‐Chi3L1 antibody compared with control mice. Similar results were obtained in the lung tissues of the metastatic model (Fig. 6E). Compared with the control group, the anti‐Chi3L1 antibody significantly reduced pSTAT6 expression in tumor and lung tissues (Fig. 6F). These results indicate that anti‐Chi3L1 antibody inhibited M2 polarization by down‐regulating phosphorylated STAT6.

### 3.7 Anti‐Chi3L1 antibody treatment decreased plasminogen expression

**Fig. 7:**
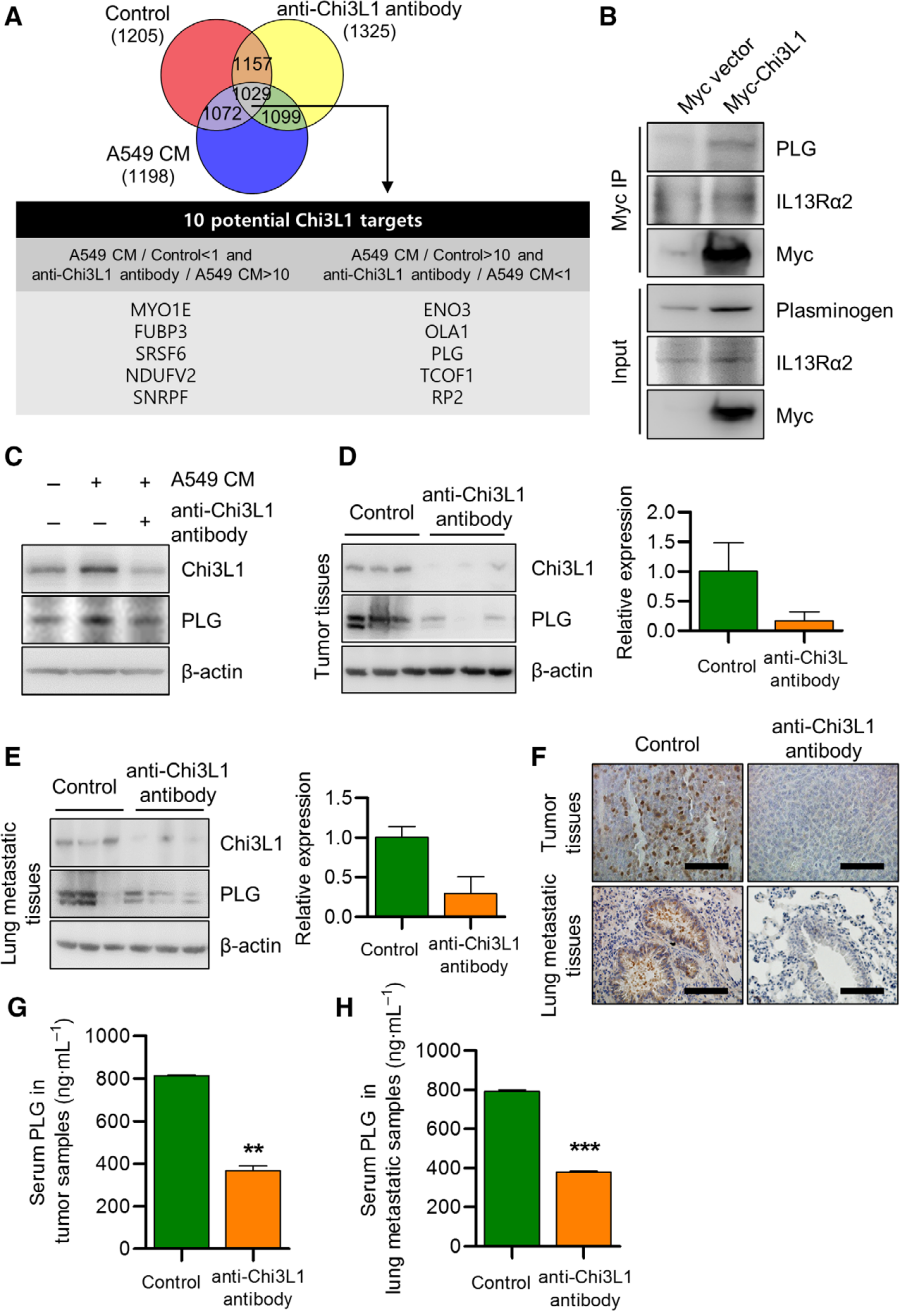
Anti‐Chi3L1 antibody treatment decreases PLG protein production and mRNA expression. (A) THP‐1 was activated using phorbol 12‐myristate 13‐acetate (PMA) and treated with A549 conditioned medium (CM). Processed peptides were subjected to nano‐high‐performance liquid chromatography (nano‐LC) and overlap between the 1029 annotated proteins was identified in THP‐1 (1881), A549 conditioned medium(CM) stimulated THP‐1 (1670, and anti‐Chi3L1 antibody‐treated THP‐1 (1804). (B) A549 cells were transfected with either Myc‐vector or Myc tagged Chi3L1 plasmids. The cell lysates were immunoprecipitated using the anti‐Myc antibody and then subjected to immunoblot analysis with the indicated antibodies. (C) THP‐1 was treated with phorbol 12‐myristate 13‐acetate (PMA) (100 ng·mL^−1^) and stimulated with A549 conditioned medium (CM) with or without anti‐Chi3L1 antibody (1 μg·mL^−1^). Western blot was performed to measure the plasminogen (PLG) protein level. (D, E) Tumor tissue and lung metastatic tissue samples were analyzed with immuno‐blotting and (F) immunohistochemistry using the antiplasminogen (PLG) antibody. Data are presented as mean ± standard deviation (SD) from three independent experiments. Scale bar, 100 μm. (G) Serum levels of plasminogen (PLG) in vehicle‐ or anti‐Chi3L1 antibody‐treated tumor tissues. Data are presented as mean ± standard deviation (SD) from three independent experiments. **P < 0.01; (unpaired two‐tailed *t*‐test). (H) Serum levels of plasminogen (PLG) in vehicle‐ or anti‐Chi3L1 antibody‐treated lung tissues. Data are presented as mean ± standard deviation (SD) from three independent experiments. ***P < 0.001; (unpaired two‐tailed *t*‐test).

To investigate potential target genes of Chi3L1, THP‐1 was stimulated with A549 CM with and without anti‐Chi3L1 antibody treatment, and cell lysates were digested. Using the nano‐high‐performance liquid chromatography (nano‐LC), we analyzed the changes in protein abundance in macrophages‐stimulated A549 CM with and without anti‐Chi3L1 antibody treatment. Our proteomics analysis indicates that 1029 proteins were observed in all samples in the independent triple experiments. We determined the protein abundance of MYO1E, FUBP3, SRSF6, NDUFV2, and SNRPF was decreased in A549 CM stimulation (fold change in expression for A549 CM versus control < 1 and anti‐Chi3L1 antibody treatment versus A549 CM stimulation > 10). ENO3, OLA1, PLG, TCOF1, and RP2 were increased in A549 CM stimulation and decreased in anti‐Chi3L1 antibody treatment (fold change in expression in A549 CM stimulation versus control > 10 and anti‐Chi3L1 antibody treatment versus A549 CM stimulation < 1) (Fig. 7A). The mRNA levels of the 10 genes were confirmed by RT‐qPCR. All of the genes (MYO1E, FUBP3, SRSF6, NDUFV2, SNRPF, ENO3, OLA1, PLG, TCOF1, and RP2) mRNA levels were increased by A549 CM stimulation, but only three genes (FOBP3, PLG, and EBI3) transcription level were reduced by anti‐Chi3L1 antibody treatment (Fig. S10A). The mRNA expression of PLG was the most significantly changed, and we selected PLG as a target protein of Chi3L1.

Plasminogen (PLG) is the precursor of plasmin (PLA) and is activated by a plasminogen activator to produce PLA [40]. PLG plays an important role in the immune response, and PLG deficiency leads to failed liver repair and wound healing and reproductive abnormalities [41, 42, 43]. Congenital plasminogen deficiency is a rare genetic disorder caused by an alteration in the PLG gene. Plasminogen activation plays an important role in the invasion and metastasis of lung, breast and brain cancer [41, 44, 45]. To determine the physical relationship between Chi3L1 and PLG, immunoprecipitation was performed using the anti‐Myc antibody. Immunoprecipitation demonstrated that Chi3L1 interacted with PLG (Fig. 7B). Significant PLG up‐regulation was observed with A549 CM, which was prominently inhibited by the anti‐Chi3L1 antibody (Fig. 7C). We quantified the amount of PLG through ELISA analysis and confirmed that the amount of PLG increased by A549 CM stimulation was reduced with anti‐Chi3L1 antibody treatment (Fig. S10B). M2 polarization was inhibited in PLG^‐/‐^ mice and PLG activity was influenced by cell polarization. To confirm the effect of PLG in STAT6 activity, we observed PLG expression in AS1517499 treatment. Similar to the anti‐Chi3L1 antibody treatment, PLG expression was decreased by AS1517499 in THP‐1 macrophages (Fig. S10C). This suggests that anti‐Chi3L1 antibody treatment reduces M2 polarization with STAT6‐dependent PLG signaling.

To confirm the inhibition of PLG by the anti‐Chi3L1 antibody in mouse tumor growth and metastasis in an *in vivo* model, immunoblot assay and immunohistochemistry were performed using anti‐PLG antibodies. PLG protein expression was significantly decreased in lung metastatic and tumor tissues of anti‐Chi3L1 antibody‐treated mice (Fig. 7D,E). Immunohistochemical staining of PLG was decreased in anti‐Chi3L1 antibody‐treated lung tumor and metastasis mice (Fig. 7F). PLG serum levels were significantly decreased in anti‐Chi3L1 antibody‐treated lung tumor mice (Fig. 7G) and metastasis mice (Fig. 7H). This anti‐Chi3L1 antibody could inhibit lung tumor growth through STAT6‐dependent PLG‐mediated Chi3L1 signal inhibition.

## 4 Discussion

Chi3L1 is highly expressed in human lung cancer patients. We demonstrated that the pharmacologic inhibition of Chi3L1 using humanized antibody anti‐Chi3L1 antibody attenuates lung tumor growth and metastatic node formation in lung cancer. Anti‐Chi3L1 antibody treatment attenuated tumor growth, metastasis in a lung cancer mouse model. We confirmed inhibition of Chi3L1 using anti‐Chi3L1 antibody inhibited M2 polarization through STAT6 activation *in vivo* and *in vitro*. A proteomics analysis revealed that PLG was identified as a novel Chi3L1 target protein. Anti‐Chi3L1 antibody inhibits tumor growth and metastasis through STAT6‐induced M2 polarization inhibition and PLG expression.

The market for protein and antibody therapeutics is growing rapidly, and expanding with the development of biopharmaceuticals, bio‐similars and bio‐betters. Antibody therapy has shown a high therapeutic effect in small amounts through its excellent targeting ability. Its developmental scope is expanding as an immunological disorder and anticancer treatment. Lung cancer is frequently diagnosed at advanced stages, is generally difficult to treat, and generates high mortality rates. Several trials have recently shown the possible advantages of antibodies and biological inhibitors in NSCLC. Avastin is a humanized antibody to VEGF, a potent angiogenesis factor that blocks signaling by inhibiting the complex formation of VEGF and VEGFR2. However, the efficacy of a single Avastin treatment remains unclear. It is currently being used in combination therapy with several chemical agents. Various chemical agents are used in combination treatment, which may cause side effects. Novel treatment strategies with new targets are needed to increase the response rate of existing treatments and reduce side effects. VEGF, PD‐L1, and CEACAM are important targets for lung cancer, and we confirmed the importance of Chi3L1 in lung cancer diagnosis. The sensitivity and specificity of serum Chi3L1 in lung cancer diagnosis are 85% and 85%, respectively. Chi3L1 expression increase as lung cancer progresses. The serum Chi3L1 analysis results of each stage of lung cancer show that the AUC value was more than 0.9. AUC values of stage 3 lung cancer patients were 1. However, the serum levels of VEGF, PD‐L1, and CEACAM did not change except for the PB‐1 levels in stage 3. These results indicate that Chi3L1 will be more useful in lung cancer diagnosis compared with other tumor markers. We have previously developed a humanized monoclonal antibody targeting Chi3L1 which significantly inhibited tumor growth and metastasis *in vivo* [25]. Injecting 0.5 mg·mL^−1^ of anti‐Chi3L1 antibody twice a week for 4 weeks significantly reduced, tumor growth and size compared with Avastin treatment. The clinically recommended dose of Avastin in NSCLC patients is 15 mg·kg^−1^. However, our *in vivo* experiments showed that Avasin and anti‐Chi3L1 antibody treated at the same dose and time showed better anticancer effects in the anti‐Chi3L1 antibody‐treated mice. The anti‐Chi3L1 antibody is an emergent anticancer drug with a low dose and administration time.

This study provides new mechanistic insights into the antitumor function of anti‐Chi3L1 antibody in a tumor microenvironment, which can regulate tumor metastasis and affect macrophage biology. Many studies have revealed that M2‐like polarization contributes to angiogenesis, cancer progression, and metastasis in various types of cancer including pancreatic cancer, liver cancer, ovarian cancer, and NSCLC [46, 47, 48, 49]. Several studies have reported on the effect of Chi3L1 on the biological functions of macrophages [35, 37, 50]. Chi3L1 regulate cytokine production including IL‐2, IFN‐β, and IL‐4 and also induce activation of the PI3K‐AKT signaling pathway [51, 52]. Chi3L1 activates ERK, Akt, and Wnt/β‐catenin signals by activation of IL‐13Rα2 signaling [53]. In addition, Chi3L1 promotes the secretion of chemokines such as IL‐8, MCP‐1, and CCL2 and plays an important role in angiogenesis [52]. These reports showed that Chi3L1 regulates tumor microenvironment, especially Chi3L1 regulates tumor progression and metastasis through regulation of macrophage recruitment and M2‐like macrophage polarization [35, 36, 37, 54]. We found that the anti‐Chi3L1 antibody efficiently suppressed the expression of prototypical M2‐marker CD206 and Arg‐1 and M2 genes in an *in vivo* model. We also confirmed that the CM from macrophages stimulated by A549 CM promoted A459 cell migrations. This migration was attenuated when the cells were incubated with the CM of THP‐1 treated with anti‐Chi3L1 antibody and A549 CM. Therefore, the anti‐Chi3L1 antibody could inhibit tumor migration through M2 polarization suppression. These results demonstrate the antitumorigenic effects of this antibody.

In this study, we confirmed that the anti‐Chi3L1 antibody has an important effect on tumorigenesis, cancer metastasis, and macrophage polarization in a lung cancer model. Interestingly, these tumor growth inhibition effects may be associated with STAT6 inhibition. STAT6 activation activates the genes responsible for M2 polarization and tumor growth [38, 39, 55]. A recent study reported that STAT6 is involved in various human diseases, through its impacts on cell differentiation, cytokine production, and cancer development and progression [56, 57, 58]. We demonstrated that the expression of M2 polarization markers such as CD206 and Arg1 was increased by activating the STAT6 signaling pathway, but its effect was significantly reduced *in vivo* and *in vitro* by anti‐Chi3L1 antibody treatment. *In vitro* studies have shown that A549CM stimulated STAT6 phosphorylation, and nuclear translocation was inhibited by anti‐Chi3L1 antibodies in macrophages. These results suggest that anti‐Chi3L1 antibody‐associated lung tumor growth inhibition might inhibit STAT6 pathway dependent M2 polarization.

To explore the molecular mechanisms behind the anti‐Chi3L1 antibody’s role in antitumorigenesis, we used quantitative proteomics to determine the targets of Chi3L1. PLG plays an important role in the invasion and metastasis of lung, breast, and brain cancer. The PLG/PLA system participates in various physiological and pathological processes, including angiogenesis, tumor growth, metastasis, and inflammatory reactions [59, 60, 61]. PLG regulates macrophage invasion, MMP proteins, or CCL2/CCR2 axis and is involved in cancer cell proliferation, migration, growth, and metastasis [62, 63]. Based on the proteomics results, the interaction between Chi3L1 and PLG was confirmed using immunoprecipitation. The increased PLG expression in A549 stimulated macrophages was reduced by anti‐Chi3L1 antibody treatment. PLG expression in lung tumor and metastatic tissues was significantly decreased in anti‐Chi3L1 antibody‐treated mice. Several studies have shown the effect of PLG on STAT4 activation rather than STAT6 activation. However, our data suggest that STAT6 inhibitor affects the expression of PLG in THP‐1 macrophages. Anti‐Chi3L1 antibody treatment reduces M2 polarization with STAT6‐dependent PLG signaling, eventually reducing tumor growth and cancer metastasis. The anti‐Chi3L1 antibody inhibited the PLG expression and M2 macrophage polarization through STAT6 signaling.

Consequently, our study suggests that the anti‐Chi3L1 antibody may regulate the tumor microenvironment in metastatic sites by affecting macrophage biology. In this paper, we propose that Chi3L1 is a good target therapeutic molecule for cancer and that anti‐Chi3L1 antibody requires clinical study.

## 5 Conclusions

This study provides the importance of anti‐Chi3L1 antibody regulating tumor growth and metastasis through STAT6‐dependent M2 polarization inhibition in lung cancer.

## Conflict of interest

The authors declare no conflict of interest.

## Author contributions

JEY designed and performed most of the experiments. IJY, DJS, JSY, and SBH assisted data interpretation. JTH supervised the entire project. JEY and JTH wrote the manuscript.

### Peer Review

The peer review history for this article is available at https://publons.com/publon/10.1002/1878‐0261.13152.

## Supporting information


**Fig. S1A‐B**. Chi3L1 plays an important role in human lung cancer.

Fig. S1C.

**Fig. S2**. Expression of Chi3L1 in lung tumor model.

**Fig. S3**. Expression of Chi3L1 in lung metastatic model.

**Fig. S4**. Anti‐Chi3L1 antibody suppresses the melanoma metastasis of lung tissues.

**Fig. S5**. Anti‐Chi3L1 antibody inhibits the expression of macrophage marker proteins in lung tumor tissues.

**Fig. S6**. Anti‐Chi3L1 antibody efficiently inhibits the M2‐like polarization of macrophages induced by IL‐4/IL‐13.

**Fig. S7**. Anti‐Chi3L1 antibody efficiently inhibits the M2‐like polarization of macrophages induced by IL‐4/IL‐13 in RAW 264.7 macrophages.

**Fig. S8**. STAT6 is involved in the anti‐Chi3L1 antibody‐induced inhibition of M2‐like macrophages polarization in RAW 264.7 cells.

**Fig. S9**. The combination anti‐Chi3L1 antibody and AS1517499 treatment has no additive effect on tumor migration.

**Fig. S10A**. The mRNA expression of putative Chi3L1 target genes and expression of PLG *in vivo* and *in vitro*.

Fig. S10B‐C.

Supplementary Material

## Data Availability

No data sets were generated or analyzed during this study.
