# Supplementary figures and images for: Anti‐Chi3L1 antibody suppresses lung tumor growth and metastasis through inhibition of M2 polarization
Source: Mol Oncol. 2021 Dec 20;16(11):2214–34. doi: 10.1002/1878-0261.13152 (PMC9168758; doi:10.1002/1878-0261.13152)

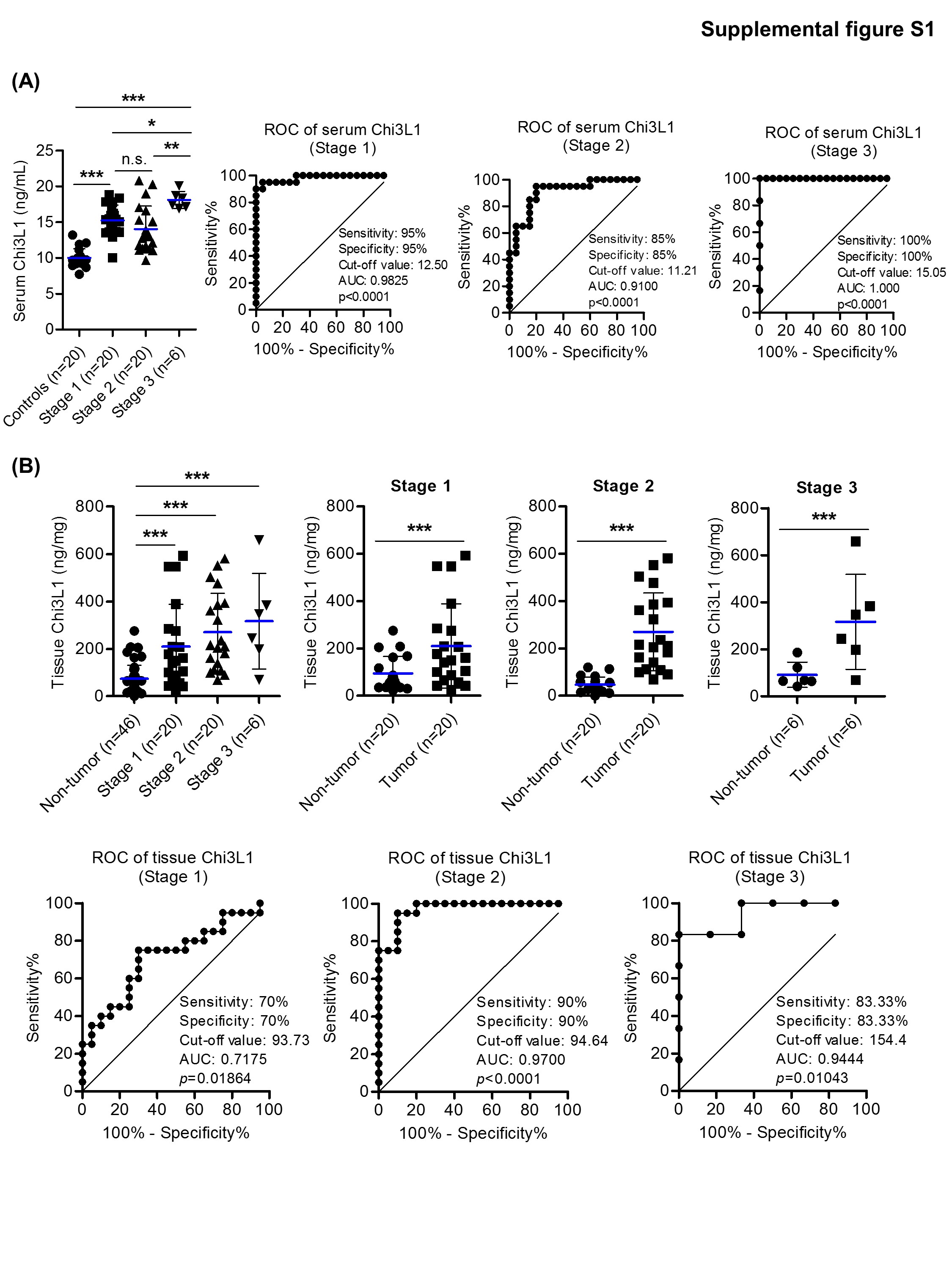

Supplement: Supplementary file 1 — Fig. S1A‐B. Chi3L1 plays an important role in human lung cancer. [file MOL2-16-2214-s011.jpeg]

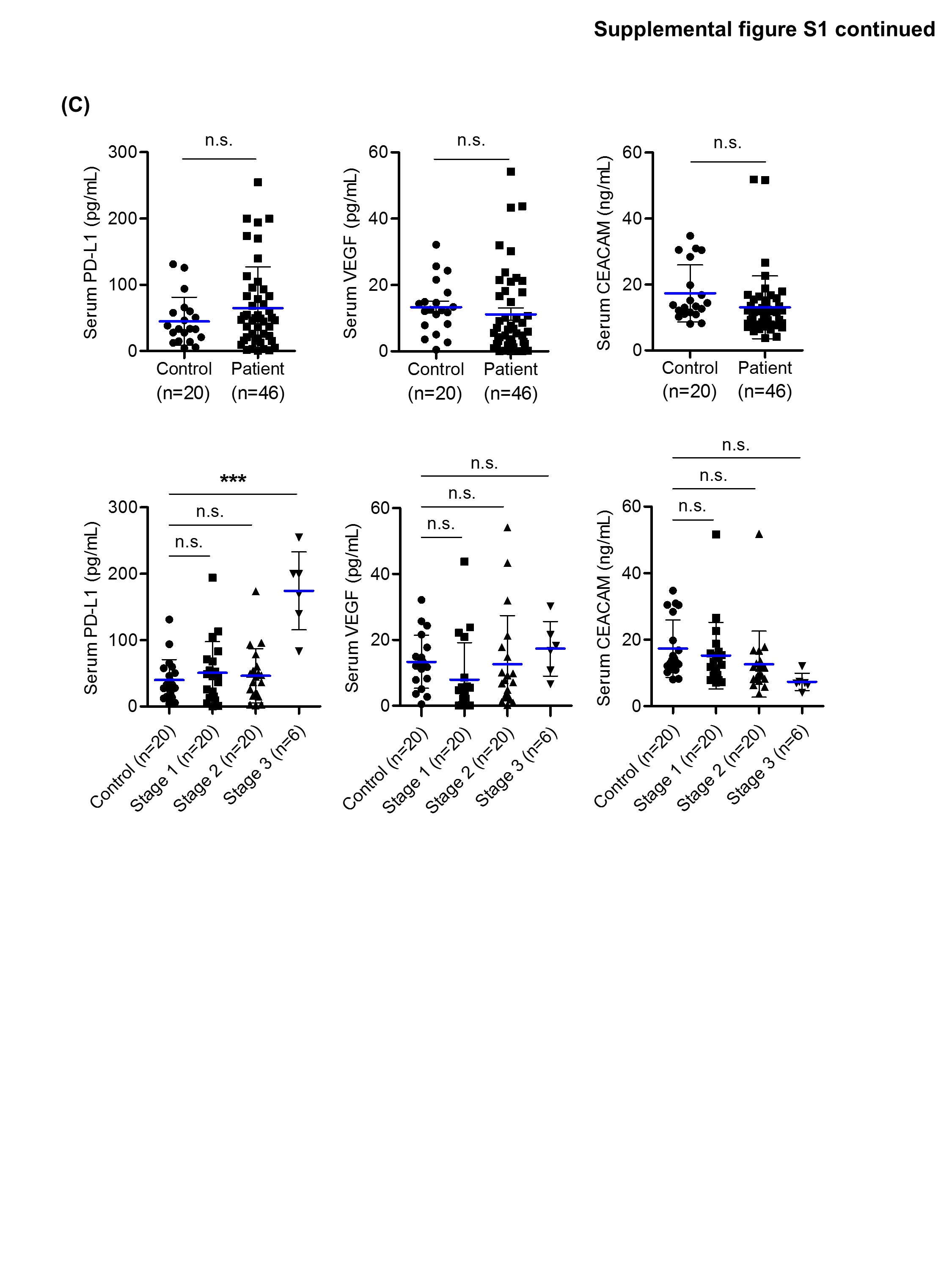

Supplement: Supplementary file 2 — Fig. S1C. [file MOL2-16-2214-s005.jpeg]

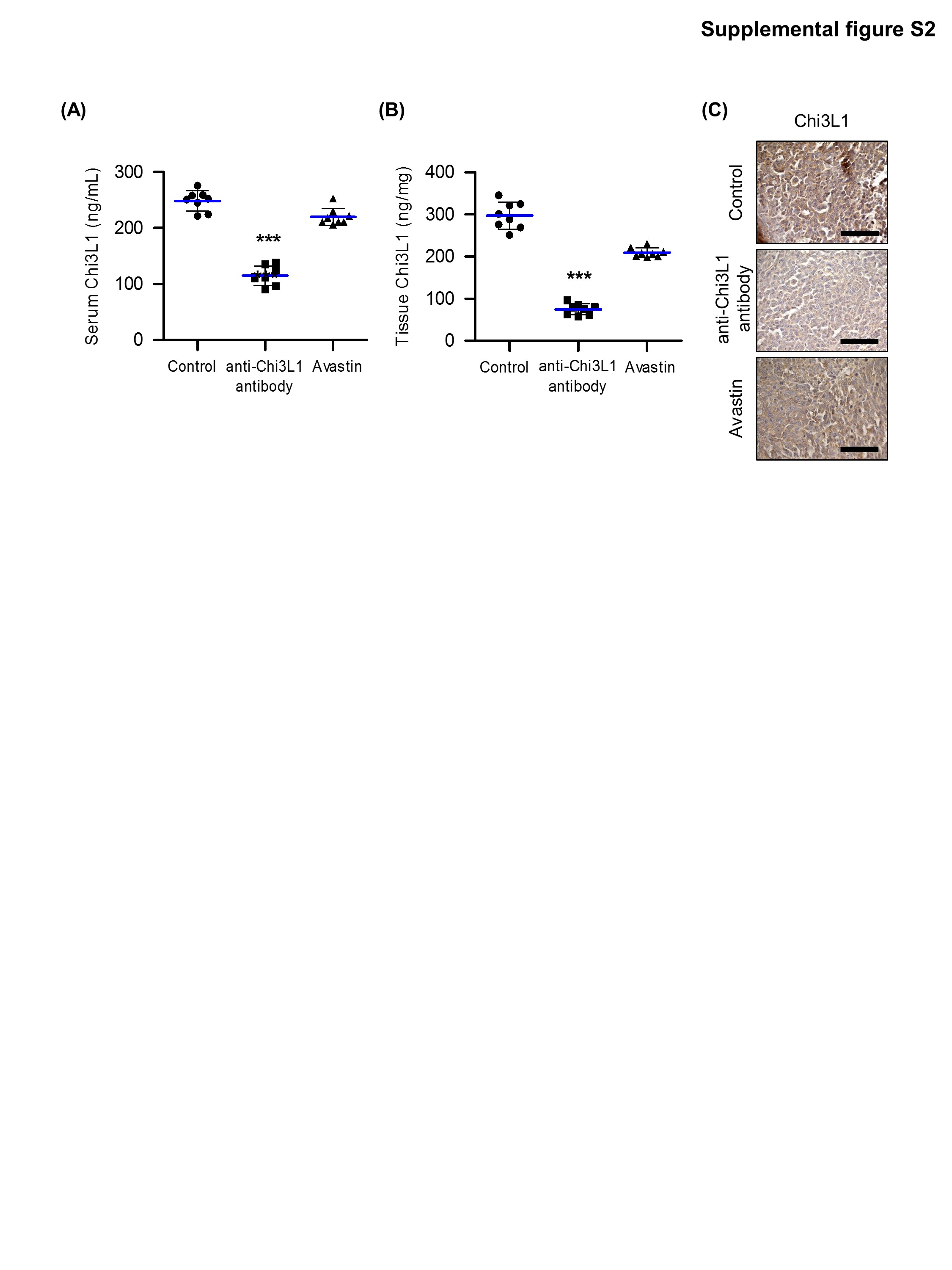

Supplement: Supplementary file 3 — Fig. S2. Expression of Chi3L1 in lung tumor model. [file MOL2-16-2214-s006.jpeg]

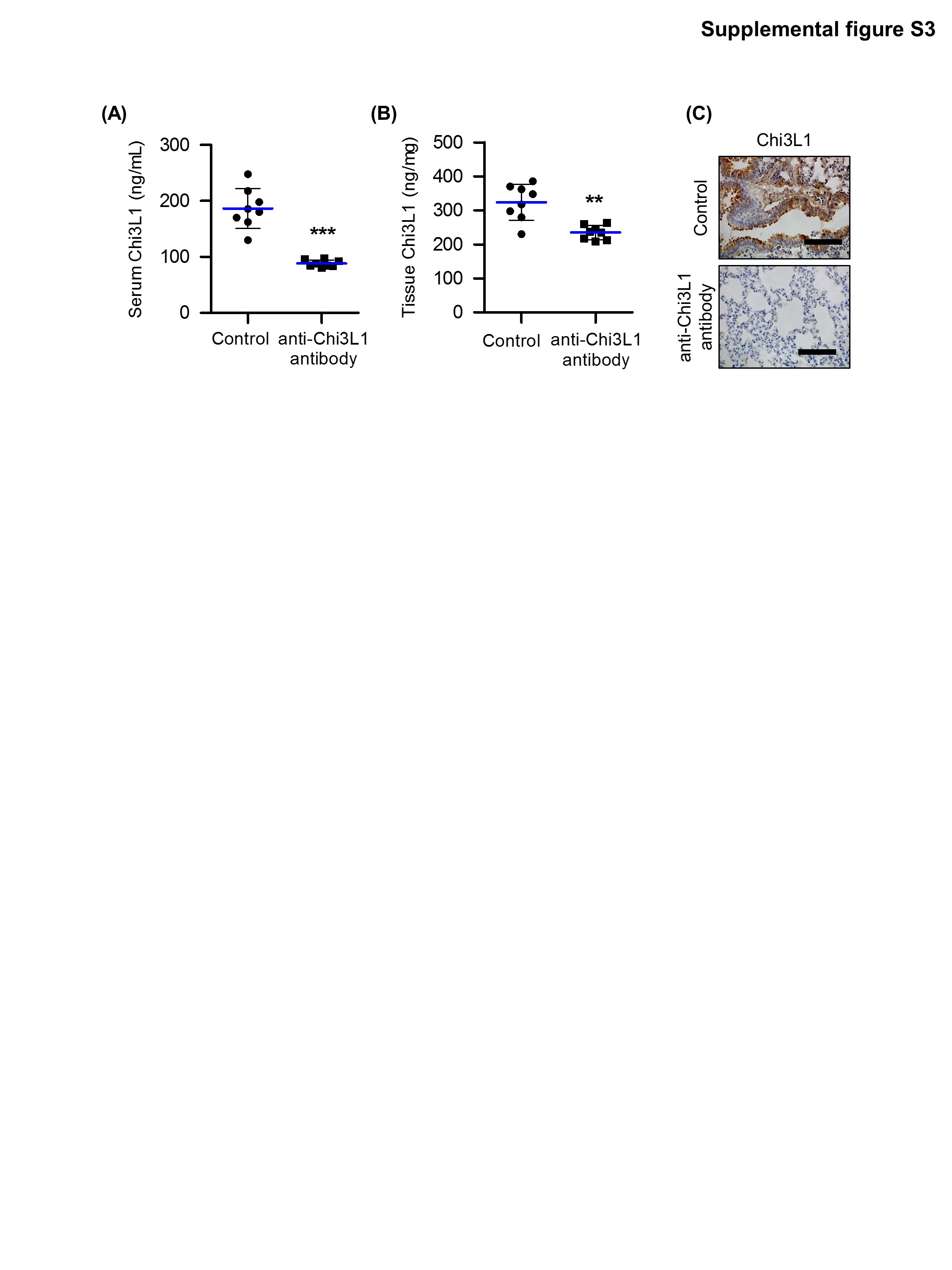

Supplement: Supplementary file 4 — Fig. S3. Expression of Chi3L1 in lung metastatic model. [file MOL2-16-2214-s003.jpeg]

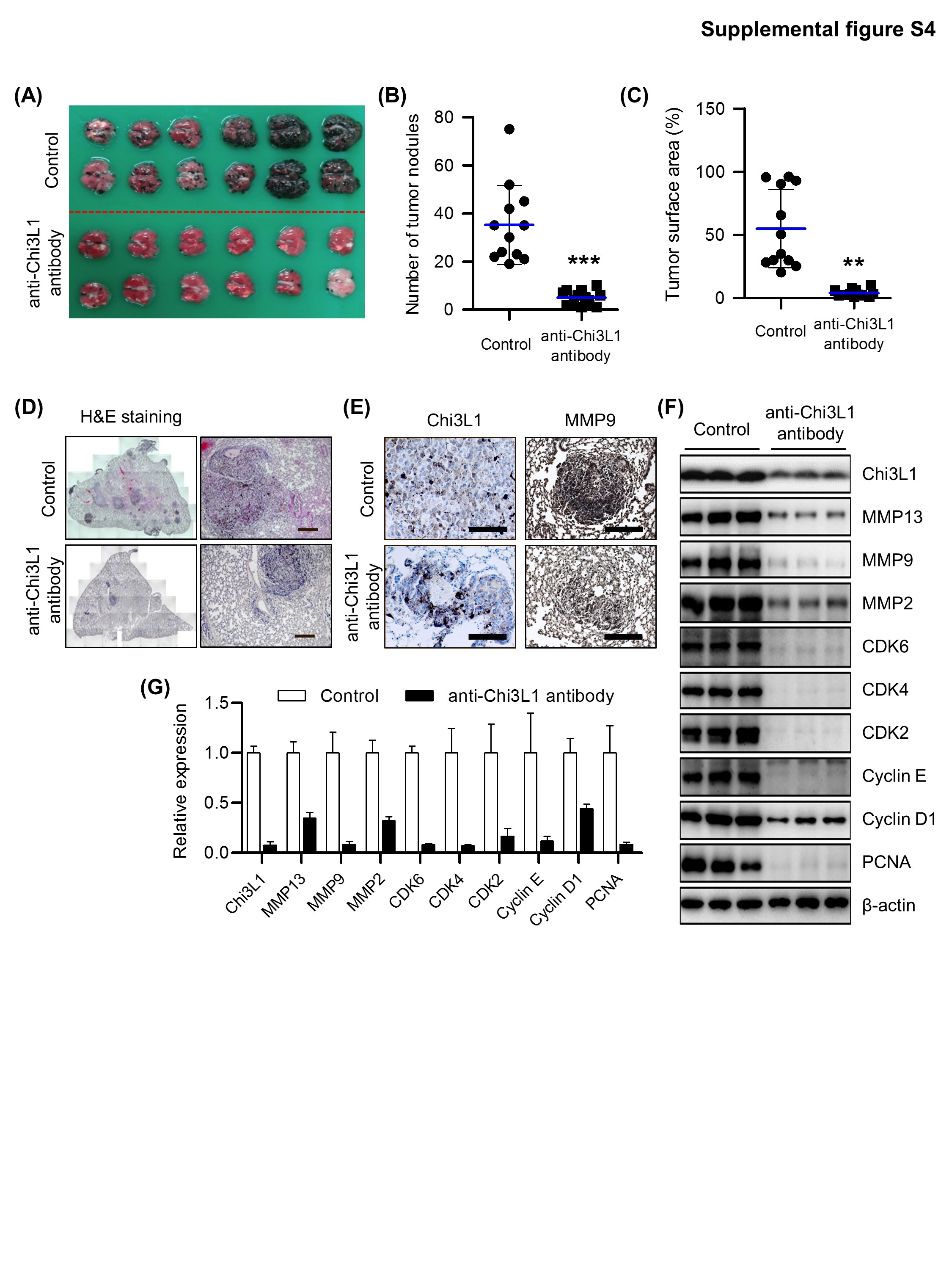

Supplement: Supplementary file 5 — Fig. S4. Anti‐Chi3L1 antibody suppresses the melanoma metastasis of lung tissues. [file MOL2-16-2214-s004.jpeg]

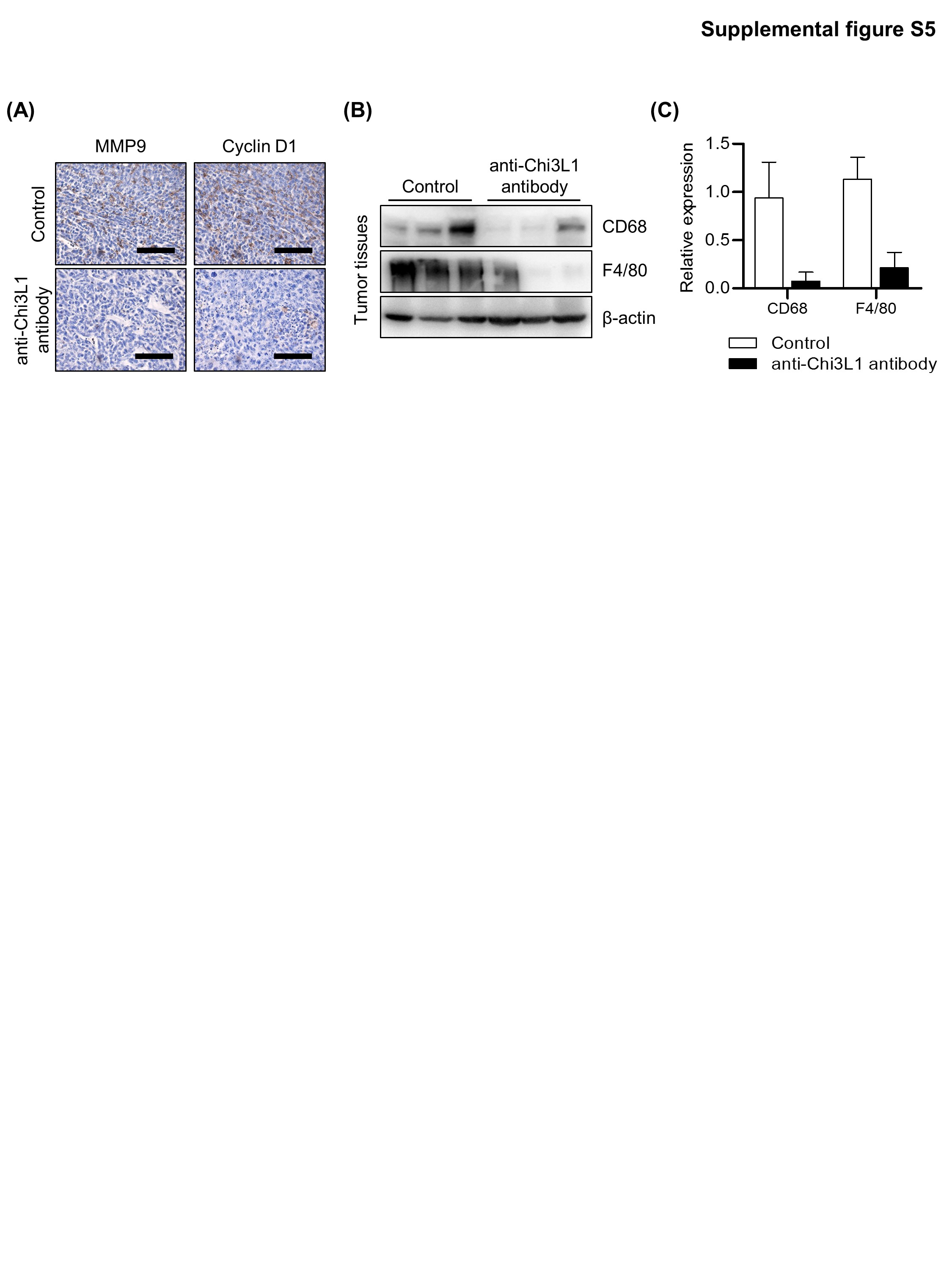

Supplement: Supplementary file 6 — Fig. S5. Anti‐Chi3L1 antibody inhibits the expression of macrophage marker proteins in lung tumor tissues. [file MOL2-16-2214-s010.jpeg]

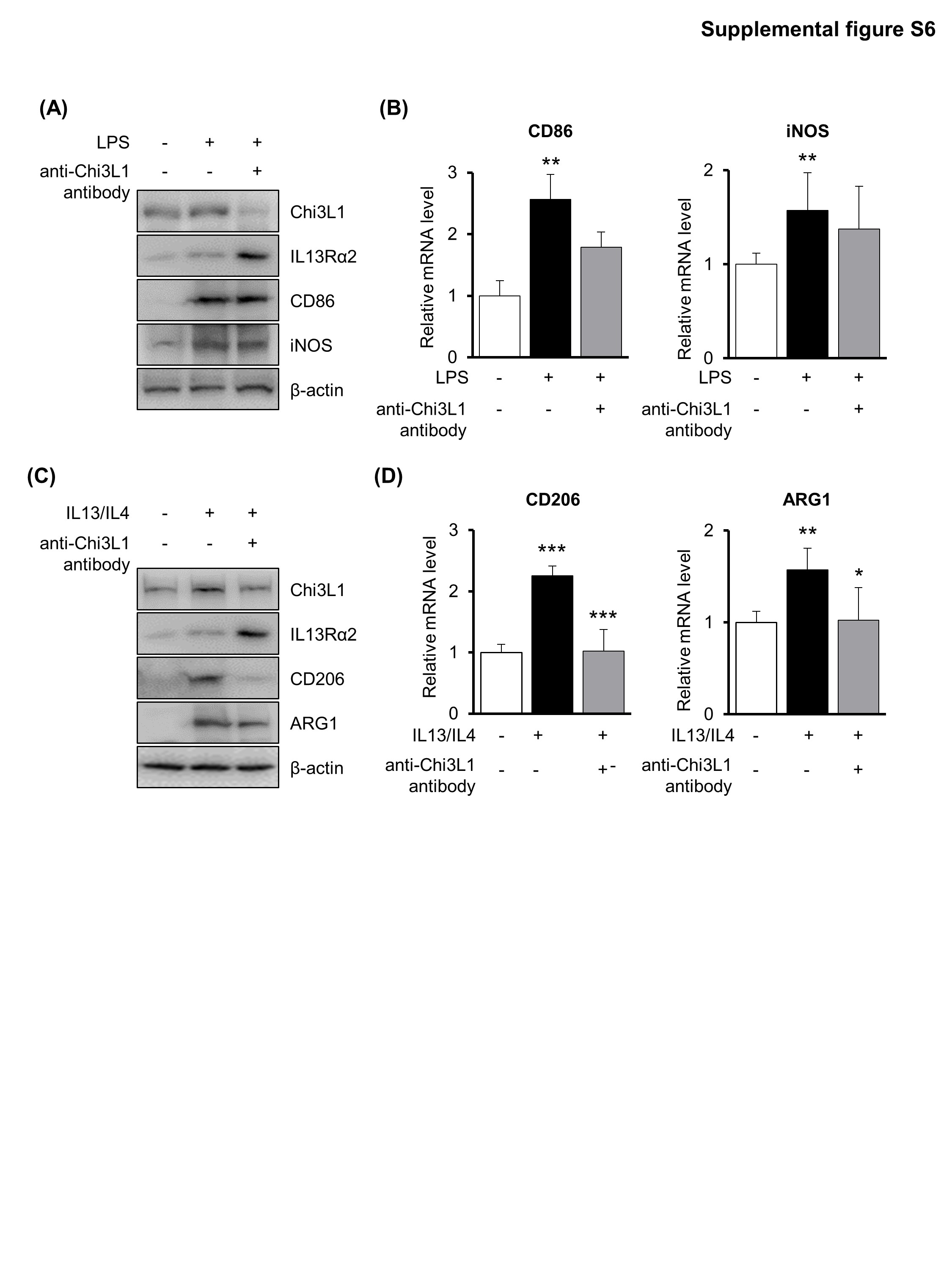

Supplement: Supplementary file 7 — Fig. S6. Anti‐Chi3L1 antibody efficiently inhibits the M2‐like polarization of macrophages induced by IL‐4/IL‐13. [file MOL2-16-2214-s009.jpeg]

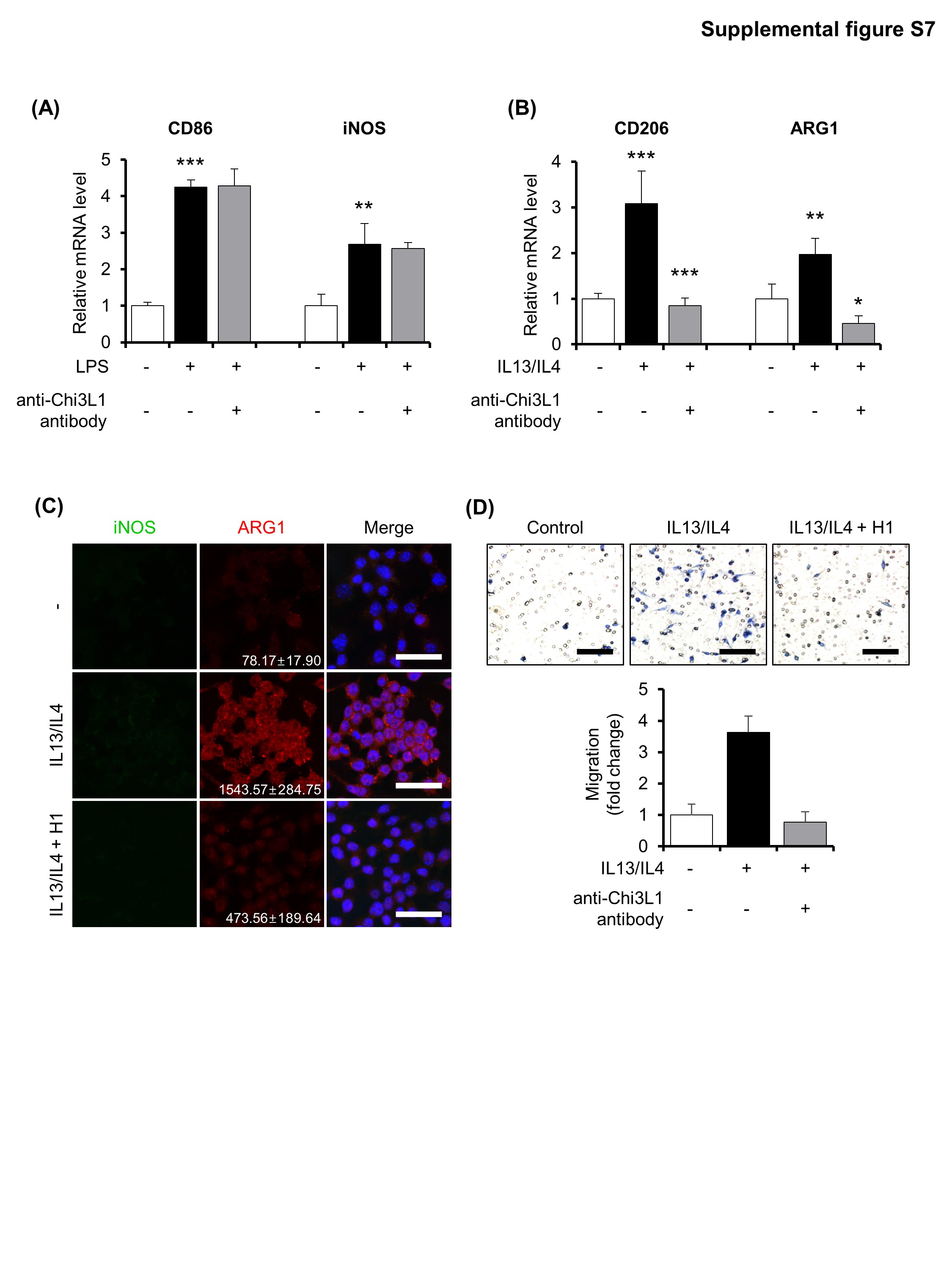

Supplement: Supplementary file 8 — Fig. S7. Anti‐Chi3L1 antibody efficiently inhibits the M2‐like polarization of macrophages induced by IL‐4/IL‐13 in RAW 264.7 macrophages. [file MOL2-16-2214-s008.jpeg]

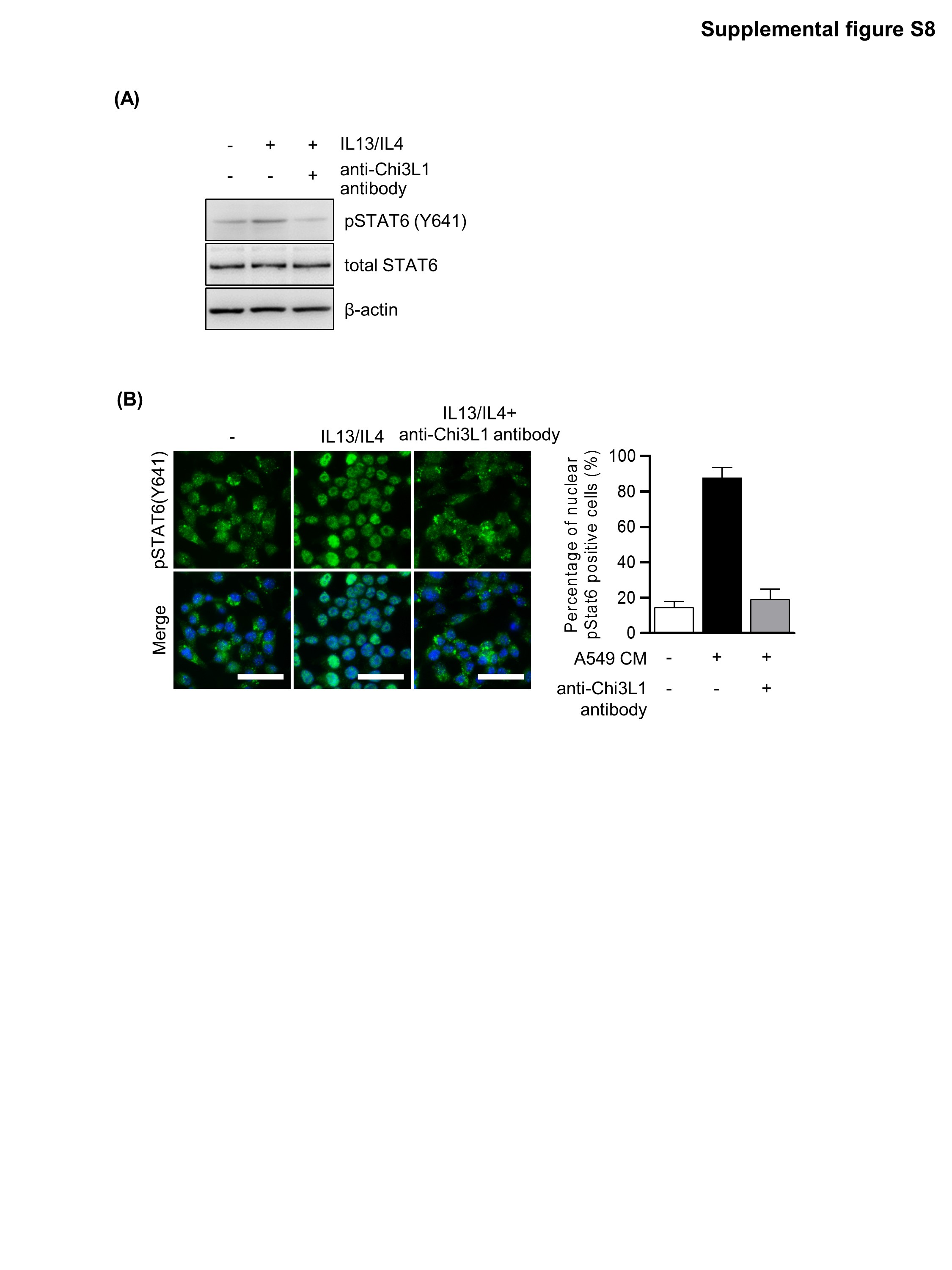

Supplement: Supplementary file 9 — Fig. S8. STAT6 is involved in the anti‐Chi3L1 antibody‐induced inhibition of M2‐like macrophages polarization in RAW 264.7 cells. [file MOL2-16-2214-s013.jpeg]

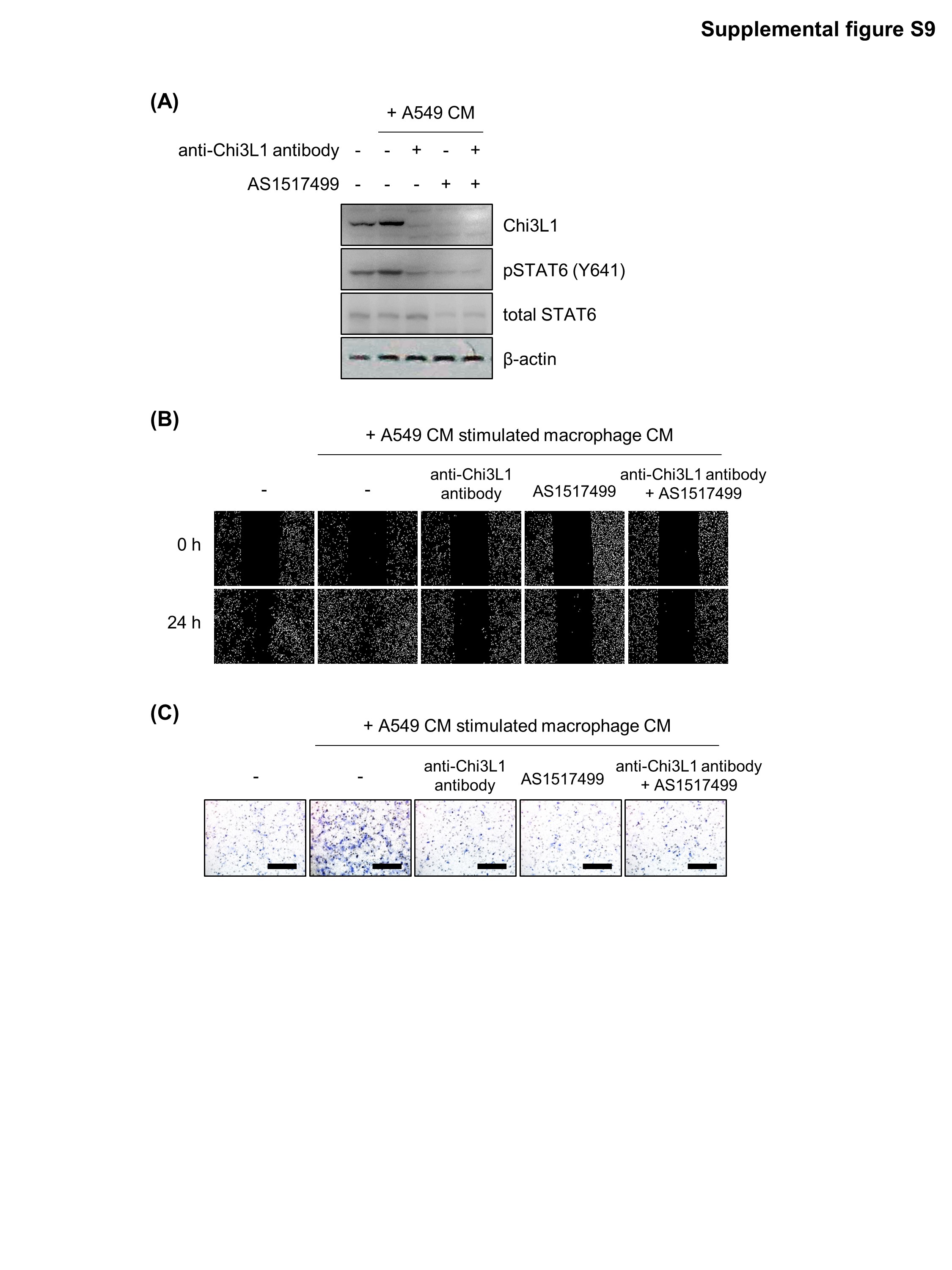

Supplement: Supplementary file 10 — Fig. S9. The combination anti‐Chi3L1 antibody and AS1517499 treatment has no additive effect on tumor migration. [file MOL2-16-2214-s001.jpeg]

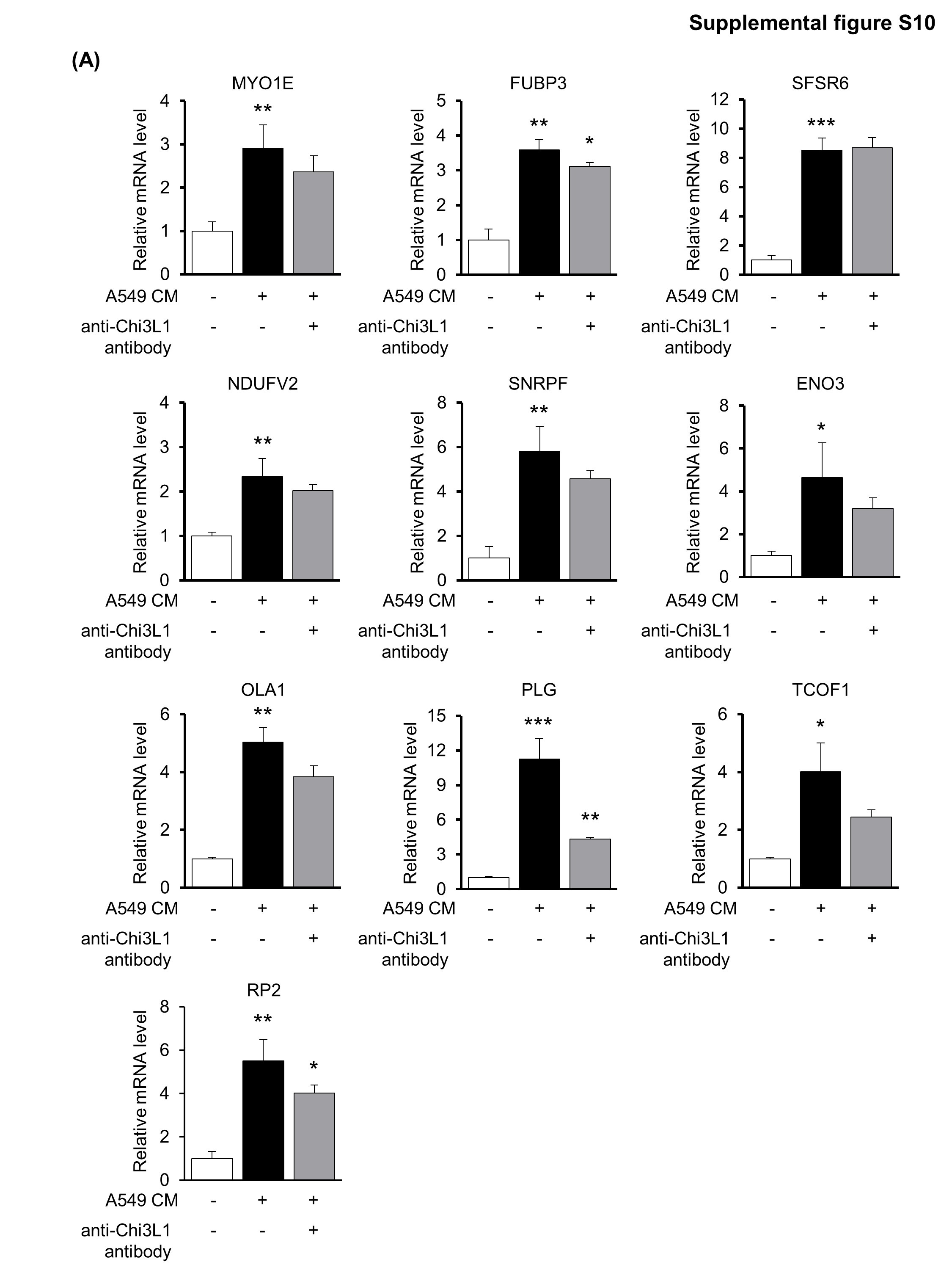

Supplement: Supplementary file 11 — Fig. S10A. The mRNA expression of putative Chi3L1 target genes and expression of PLG in vivo and in vitro. [file MOL2-16-2214-s002.jpeg]

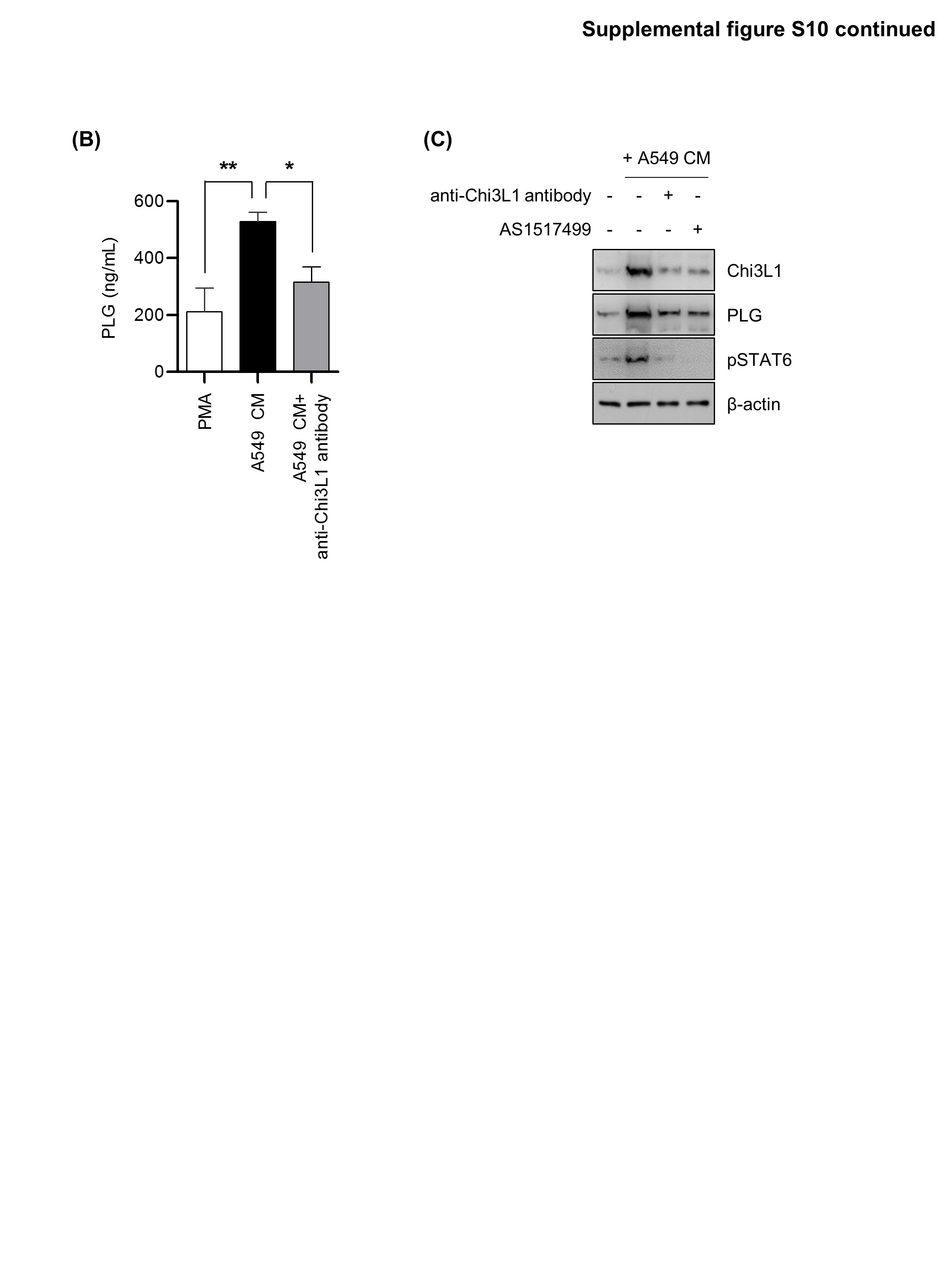

Supplement: Supplementary file 12 — Fig. S10B‐C. [file MOL2-16-2214-s012.jpeg]
