# Supplementary material for: Anti‐Chi3L1 antibody suppresses lung tumor growth and metastasis through inhibition of M2 polarization
Source: Mol Oncol. 2021 Dec 20;16(11):2214–34. doi: 10.1002/1878-0261.13152 (PMC9168758; doi:10.1002/1878-0261.13152)
Supplement: Supplementary file 13 — Supplementary Material [file MOL2-16-2214-s007.docx]

**Supporting information**

**Anti-Chi3L1 antibody suppresses lung tumor growth and metastasis through inhibition of M2 polarization**

**Ji Eun Yu^1^, In Jun Yeo^1^, Dong Ju Son^1^, Jaesuk Yun^1^, Sang-Bae Han^1^, Jin Tae Hong^1,*^**

^1^College of Pharmacy and Medical Research Center, Chungbuk National University, 194-31, Osongsaengmyeong 1-ro, Osong-eup, Cheongju-si, Chungbuk 28160, Republic of Korea.

*** *Corresponding author*:**

Dr. Jin Tae Hong

College of Pharmacy and Medical Research Center, Chungbuk National University

194-21 Osongsaengmyeong 1-ro, Osong-Biocampus, Osong-eup, Heungdeok-gu, Cheongju, Chungbuk 28160, Korea

Phone: +82-43-261-2813, FAX: +82-43-268-2732, E-mail: jinthong@chungbuk.ac.kr

**Supplemental figure legends**

**
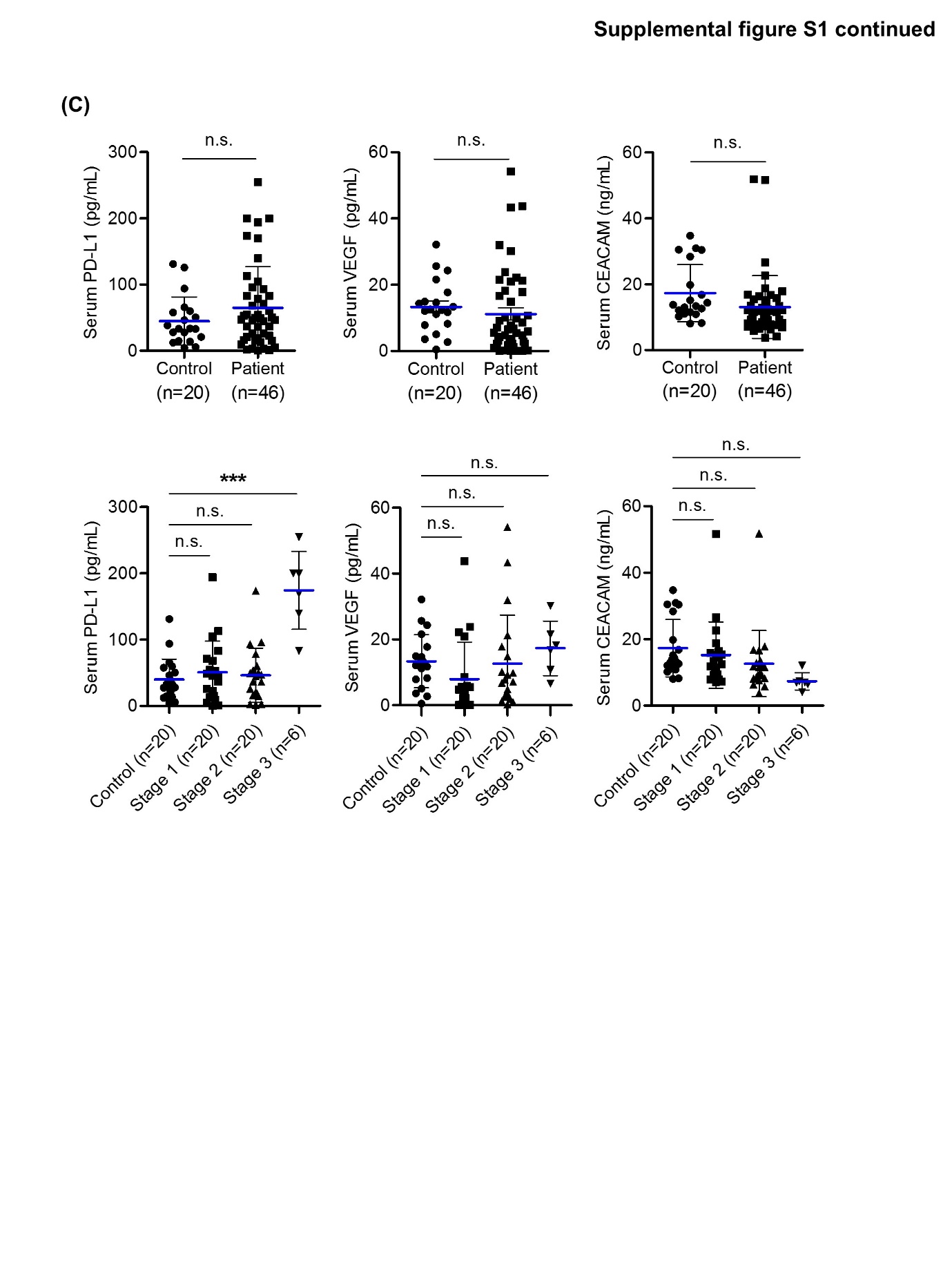
**


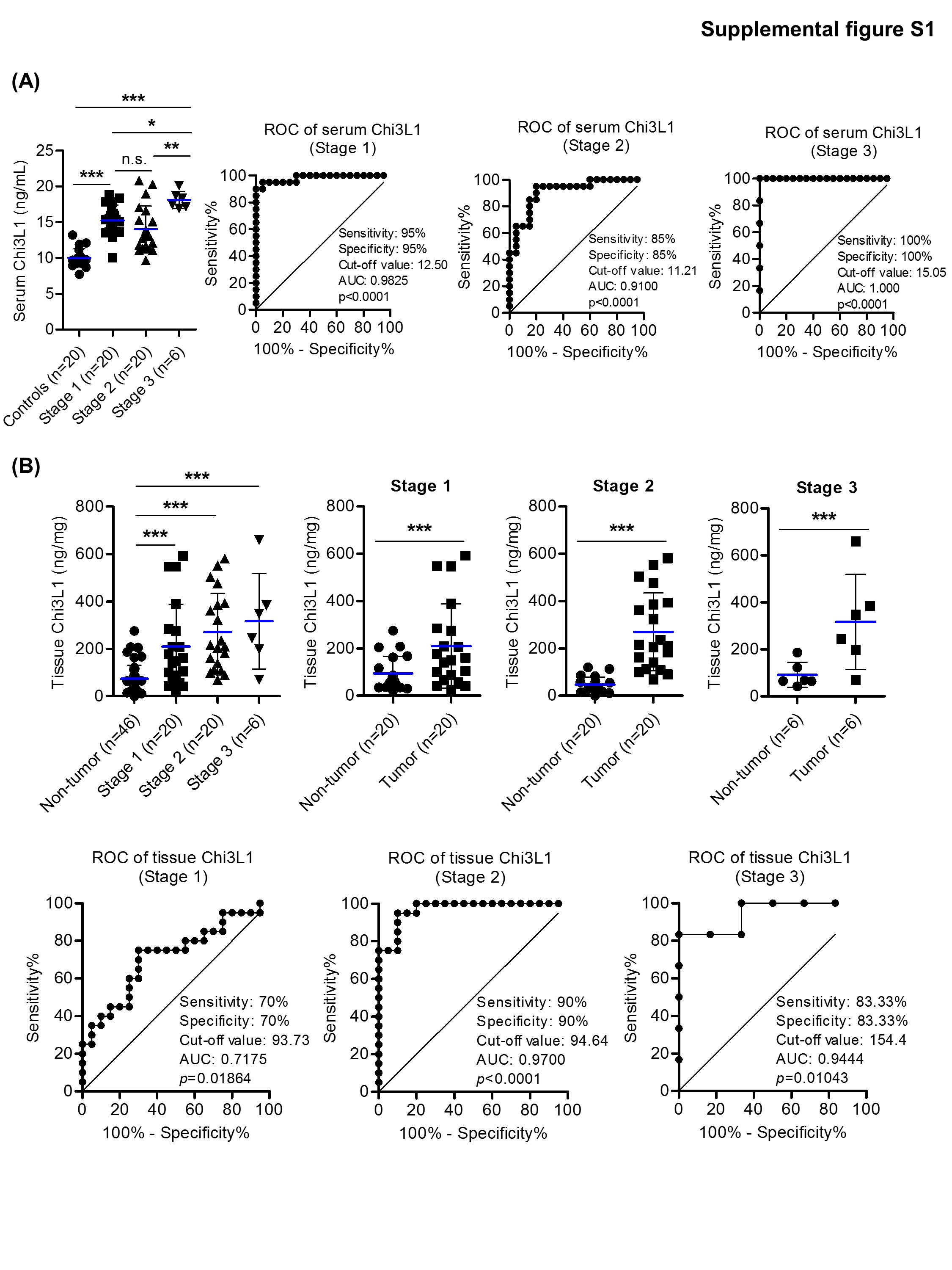


**Supplemental figure S1. Chi3L1 plays an important role in human lung cancer.**

(A) Serum levels and ROC curve of Chi3L1 at each stage of lung cancer patients and healthy controls. *, P<0.05; **, P<0.01; ***, P<0.001; n.s., P>0.05. (B) Tissue levels and ROC curve of Chi3L1 at each stage of lung cancer patients (Stage 1 non-tumor site n=20, tumor site n=20; Stage 2 non-tumor site n=20, tumor site n=20; Stage 3 non-tumor site n=6, tumor site n=6). ***, P<0.001. (C) Serum levels and ROC curve of VEGF, PD-L1, and CEACAM in lung cancer patients and healthy controls. ***, P<0.001; n.s., P>0.05.

**Supplemental figure S2. Expression of Chi3L1 in lung tumor model.**


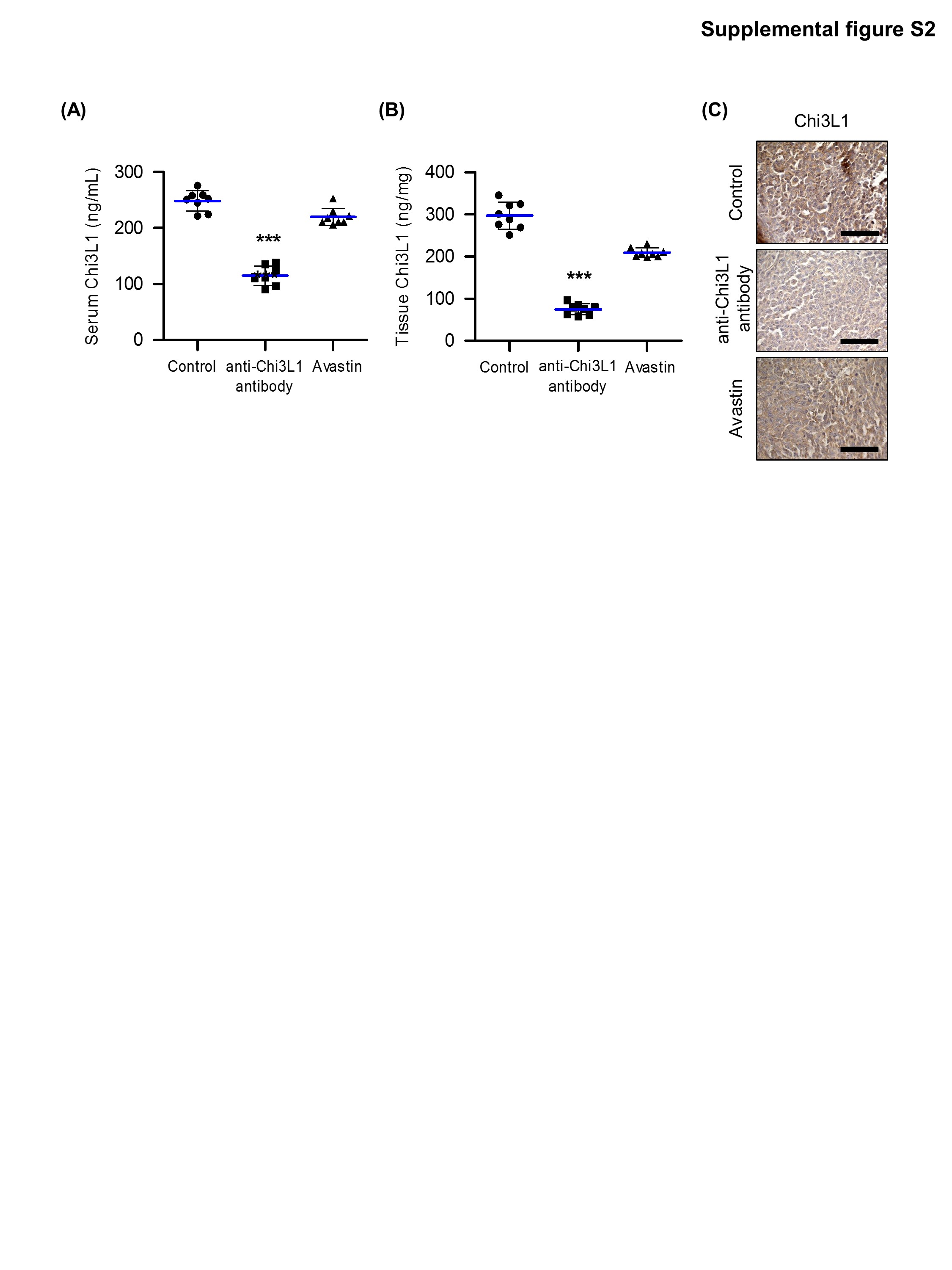


(A-C) LLC cells were injected subcutaneously to induce lung cancer tumors. PBS or anti-Chi3L1 antibody was injected intravenously twice a week for four weeks. (A) Serum levels of Chi3L1 in PBS, anti-Chi3L1 antibody or Avastin treated tumor tissues (n=8). ***, P<0.001. (B) Tissue levels of Chi3L1 in PBS, anti-Chi3L1 antibody or Avastin treated tumor tissues (n=8). ***, P<0.001. (C) Representative immunohistochemical images of tumor tissues using anti-Chi3L1 antibodies in each group. Scale bar, 100 μm.

**Supplemental figure S3. Expression of Chi3L1 in lung metastatic model.**


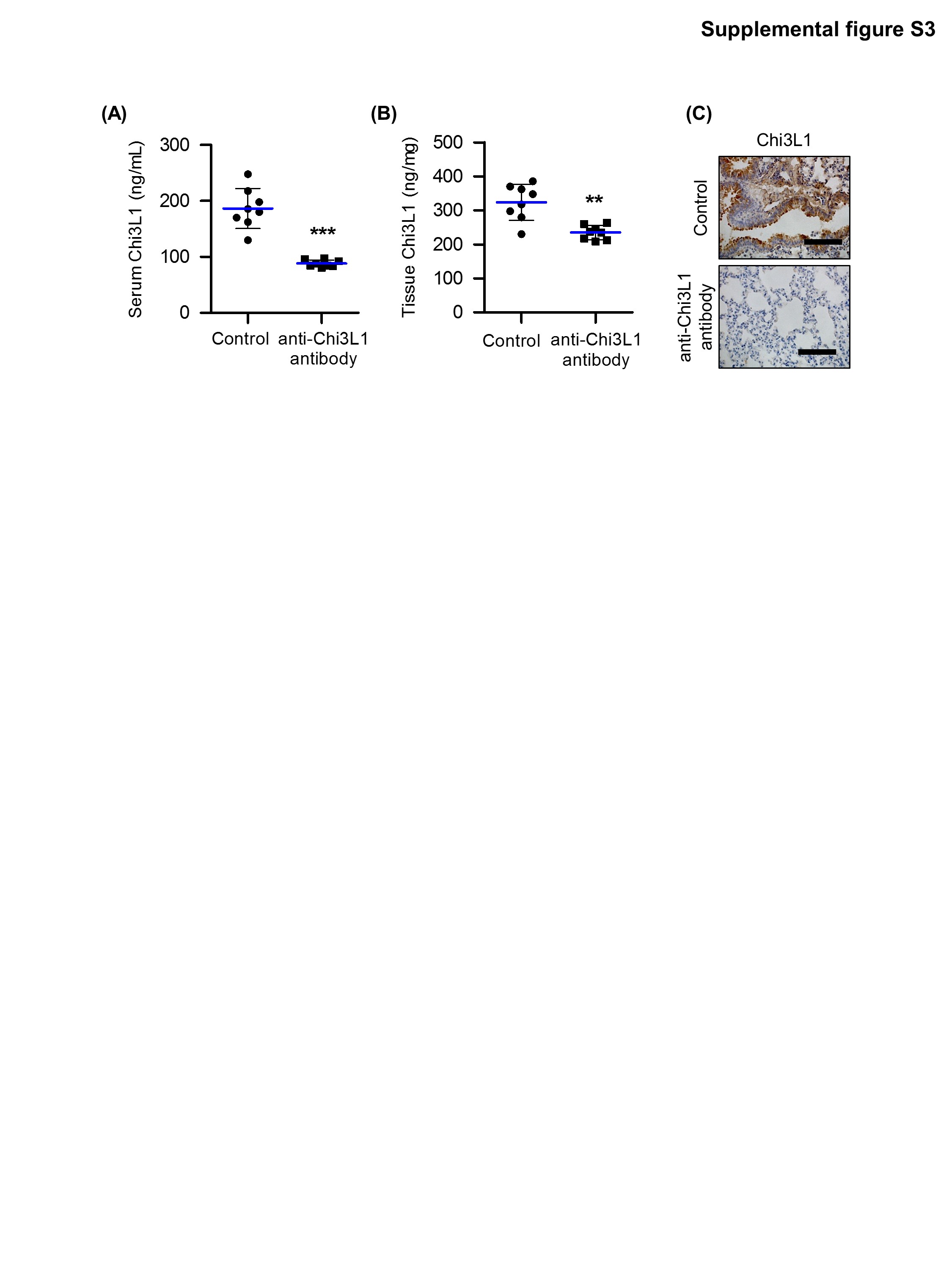


(A-C) A549 cells were injected intravenously and anti-Chi3L1 antibody were injected intravenously twice a week for eight weeks. (A) Serum levels of Chi3L1 in PBS or anti-Chi3L1 antibody-treated lung tissues (n=8). ***, P<0.001. (B) Tissue levels of Chi3L1 in PBS or anti-Chi3L1 antibody-treated lung tissues (n=8). **, P<0.01. (C) Representative immunohistochemical images of lung tissues using anti-Chi3L1 antibodies in each group. Scale bar, 100 μm.


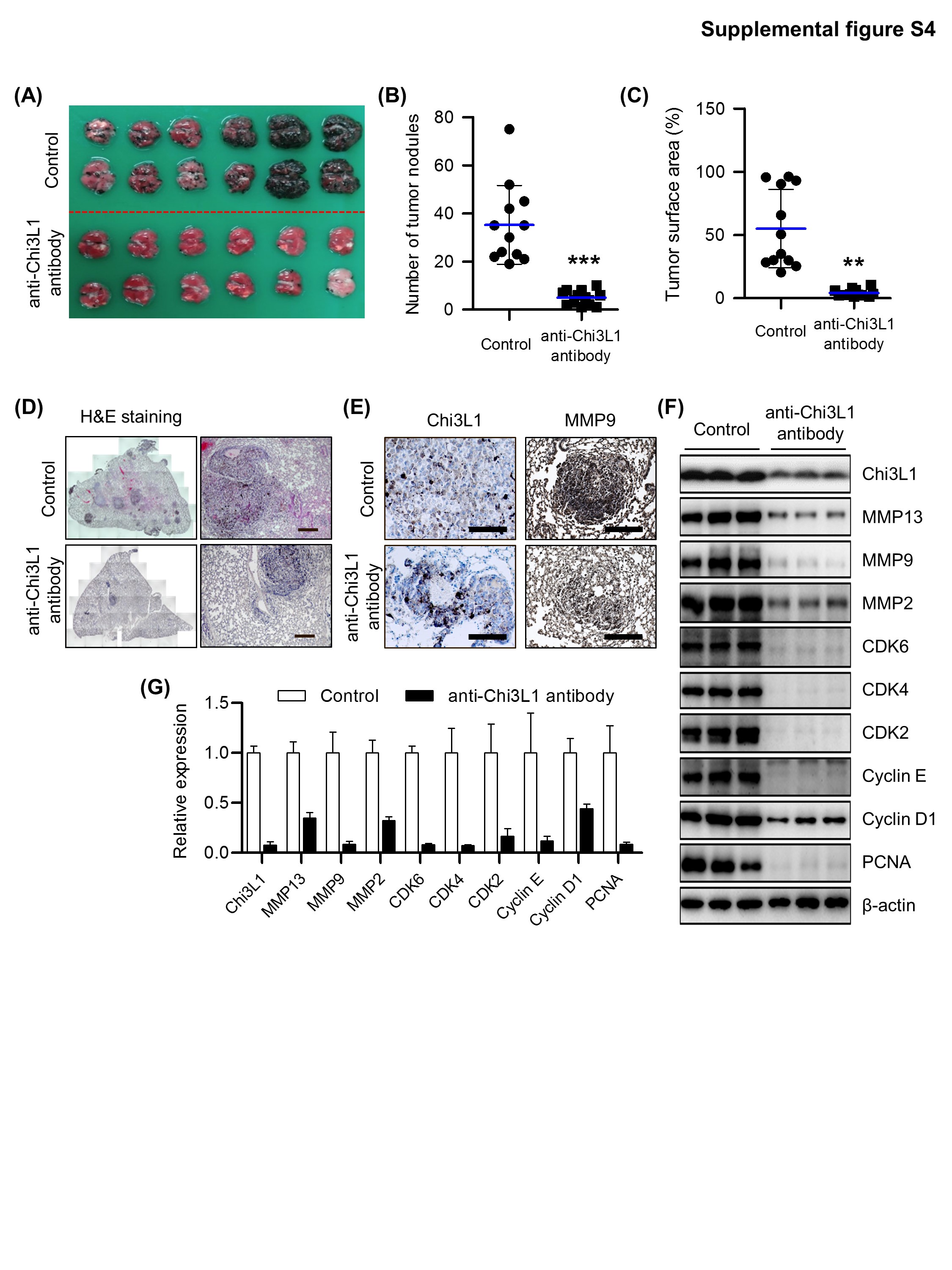


**Supplemental figure S4. Anti-Chi3L1 antibody suppresses the melanoma metastasis of lung tissues.**

B16-F10 A549 cells were injected intravenously and anti-Chi3L1 antibody were injected intravenously twice a week for eight weeks. (A) Representative image of lung metastasis progression obtained from mice in each group. (B) The number of metastatic nodules on lung surface were counted and quantified (n=12). ***, P<0.001. (C) The tumor surface areas on the lung tissue were measured from the H&E staining iamges and quantified as a percentage of total lung surface area (n=12). **, P<0.01. (D) H&E staining images of metastatic lung tissues excised from each group. Scale bar, 100 μm. (E) Representative immunohistochemical images of lung tissues using the anti-Chi3La, MMP9, and cyclin D1 antibodies in each group. Scale bar, 100 μm. (F) The lung tissue extracts were subjected to immunoblot analysis with indicated antibodies. (G) The intensity of each band in (F) was measured and the ratio of the amount of each protein to β-actin was calculated.


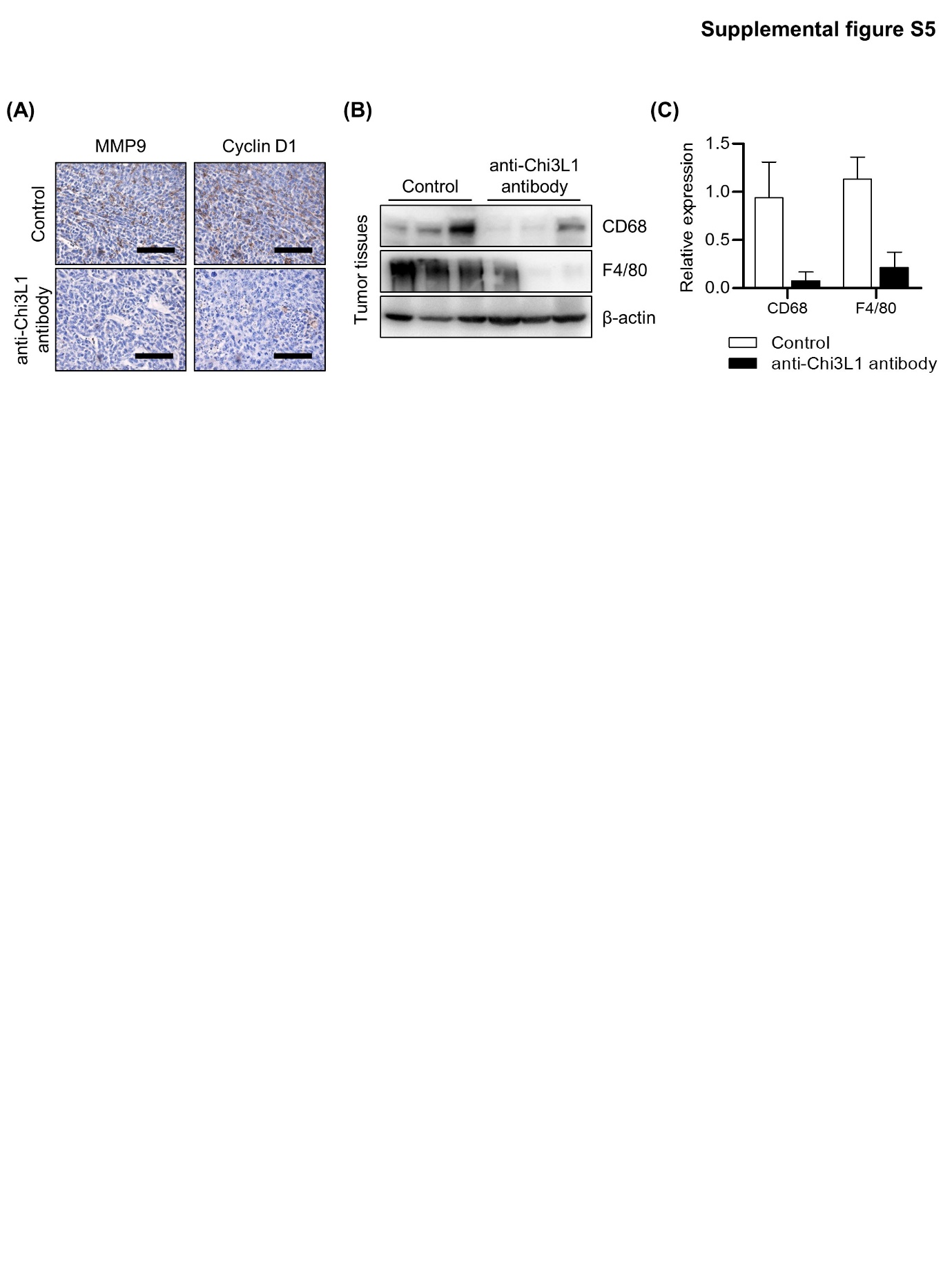


**Supplemental figure S5. Anti-Chi3L1 antibody inhibits the expression of macrophage marker proteins in lung tumor tissues.**

(A-B) LLC cells were injected subcutaneously to induce lung cancer tumors. PBS or anti-Chi3L1 antibody was injected intravenously twice a week for four weeks. (A) Representative immunohistochemical images of tumor tissues using the anti-CD68 and anti-F4/80 antibodies in each group. Scale bar, 100 μm. (B) The tumor tissue extracts were subjected to immunoblot analysis with indicated antibodies. (C) The intensity of each band in (B) was measured and the ratio of the amount of each protein to β-actin was calculated.


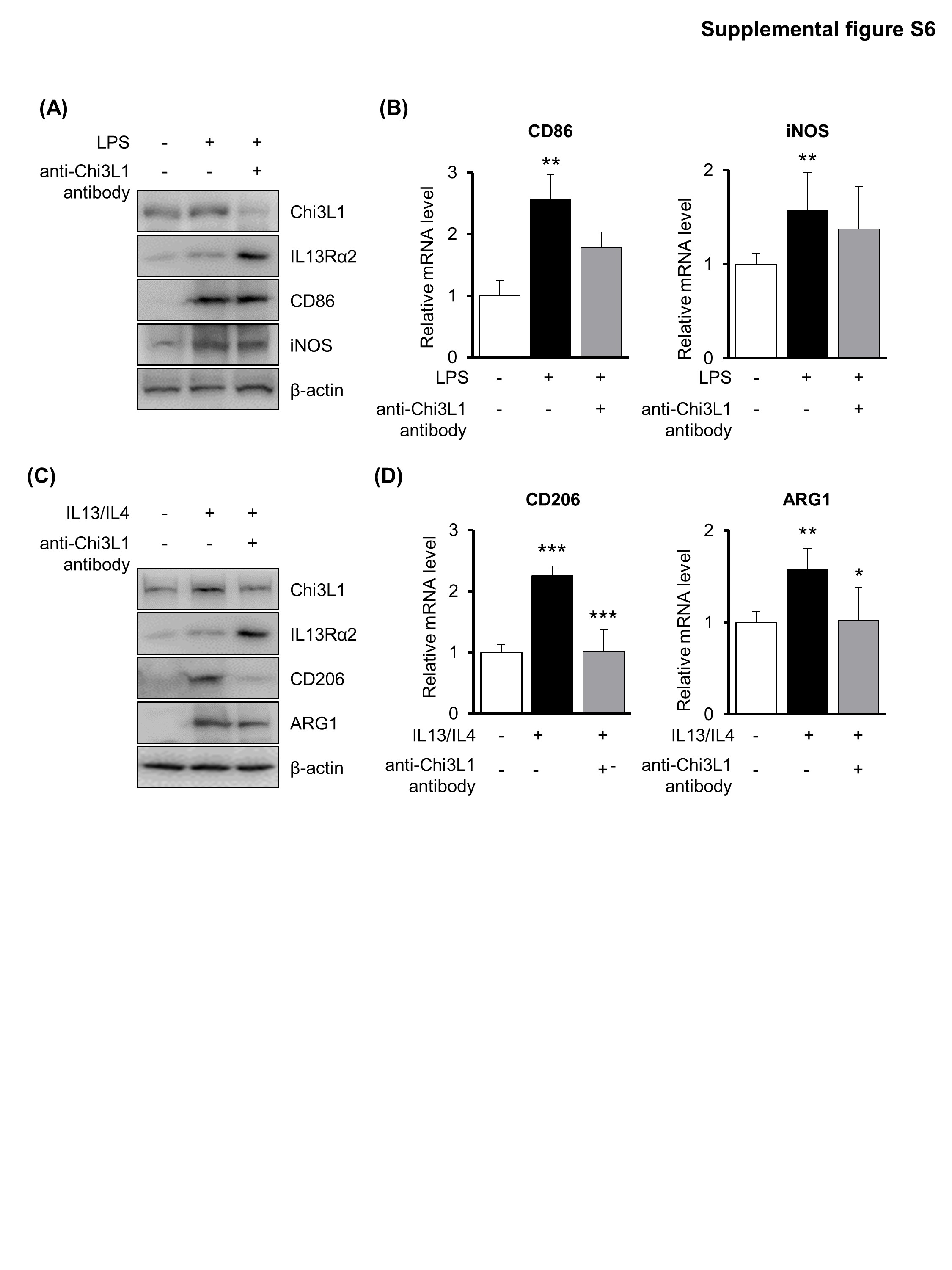


**Supplemental figure S6. Anti-Chi3L1 antibody efficiently inhibits the M2-like polarization of macrophages induced by IL-4/IL-13.**

(A, B) Thp-1 cells were treated with PMA (100 ng/ml) and stimulated with LPS (100 ng/ml) with or without anti-Chi3L1 antibody (1 μg/ml). (A) The cell lysates were subjected to immunoblot analysis with M1-marker proteins antibodies. (B) RT-qPCR analysis of M1-marker gene mRNA levels. Data are presented as mean ±SD from three independent experiments. **, P<0.01. (C, D) Thp-1 cells were treated with PMA (100 ng/ml) and stimulated with IL-4 (20 ng/ml), IL-13 (20 ng/ml) with or without anti-Chi3L1 antibody (1 μg/ml). (C) The cell lysates were subjected to immunoblot analysis with M2-marker proteins antibodies. (D) RT-qPCR analysis of M2-marker gene mRNA levels. Data are presented as mean ±SD from three independent experiments. *, P<0.05; **, P<0.01; ***, P<0.001.


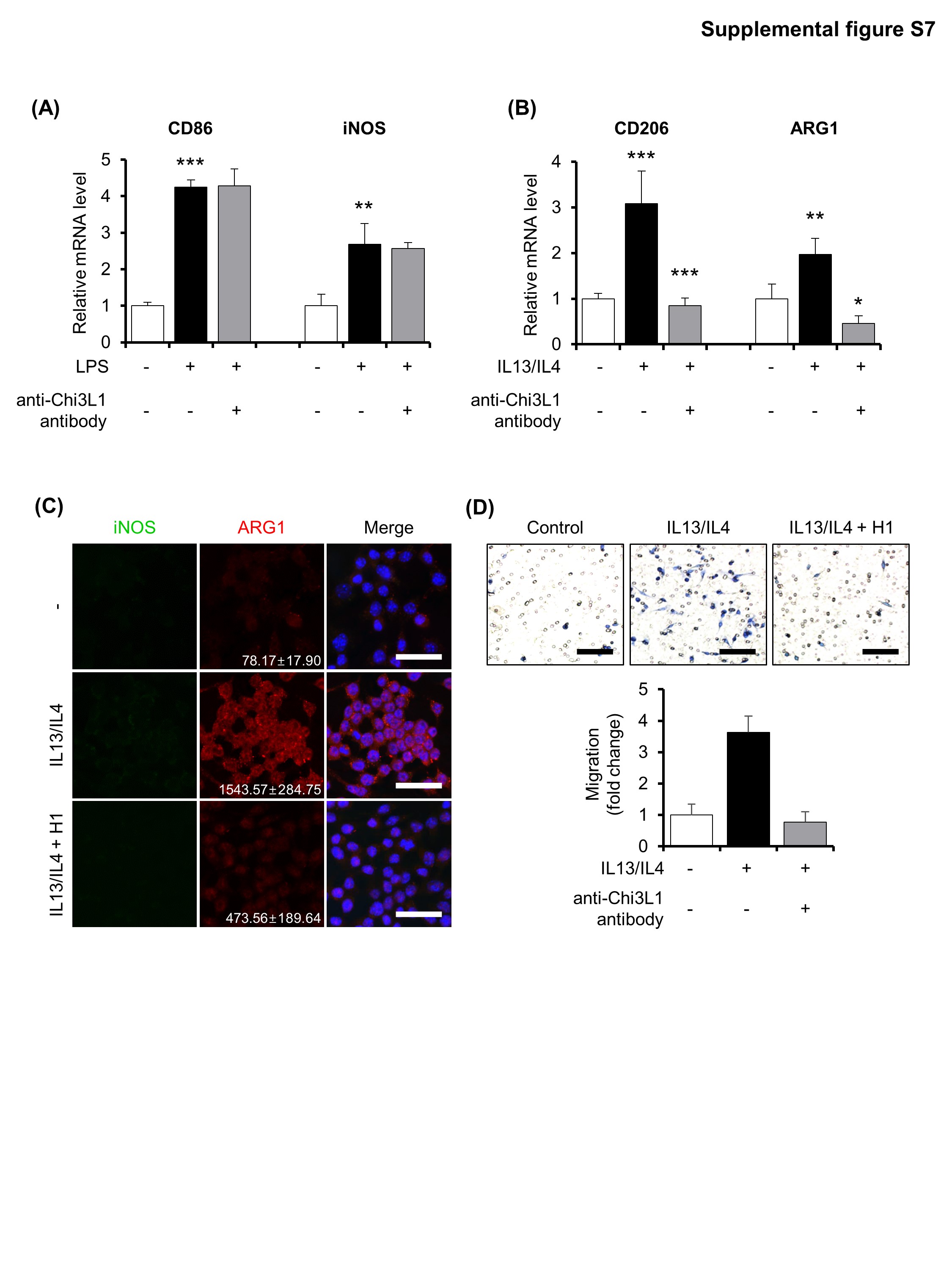


**Supplemental figure S7. Anti-Chi3L1 antibody efficiently inhibits the M2-like polarization of macrophages induced by IL-4/IL-13 in RAW 264.7 macrophages.**

(A) RAW 264.7 macrophages were stimulated with LPS (100 ng/ml) with or without anti-Chi3L1 antibody (1 μg/ml). RT-qPCR analysis of M1-marker gene mRNA levels. Data are presented as mean ±SD from three independent experiments. **, P<0.01; ***, P<0.001. (B) RAW 264.7 macrophages were stimulated with IL-4 (20 ng/ml), IL-13 (20 ng/ml) with or without anti-Chi3L1 antibody (1 μg/ml). RT-qPCR analysis of M2-marker gene mRNA levels. Data are presented as mean ±SD from three independent experiments. *, P<0.05; **, P<0.01; ***, P<0.001. (C) RAW 264.7 macrophages were stimulated with IL-4 (20 ng/ml), IL-13 (20 ng/ml) with or without anti-Chi3L1 antibody (1 μg/ml). The 4% paraformaldehyde fixed cells were immunofluorescence stained with anti-iNOS and anti-ARG1 antibodies. The fluorescence intensity of ARG1^+^ cells was measured and the figures were written in the figures. Data are presented as mean ±SD from three independent experiments. Scale bar, 50 μm. (D) The effect of anti-Chi3L1 antibody on RAW 264.7 macrophages migration was evaluated by a Trans-well assay. The RAW 264.7 macrophages were seeded in the upper chamber and IL13/IL4 treated-medium or IL13/IL4 with anti-Chi3L1 antibody treated-medium placed into the lower chambers and incubated at 37 °C for 18 h. The migrated cells on the bottom chamber were stained with 0.1% crystal violet. Data are presented as mean ±SD from three independent experiments. Scale bar, 100 μm.


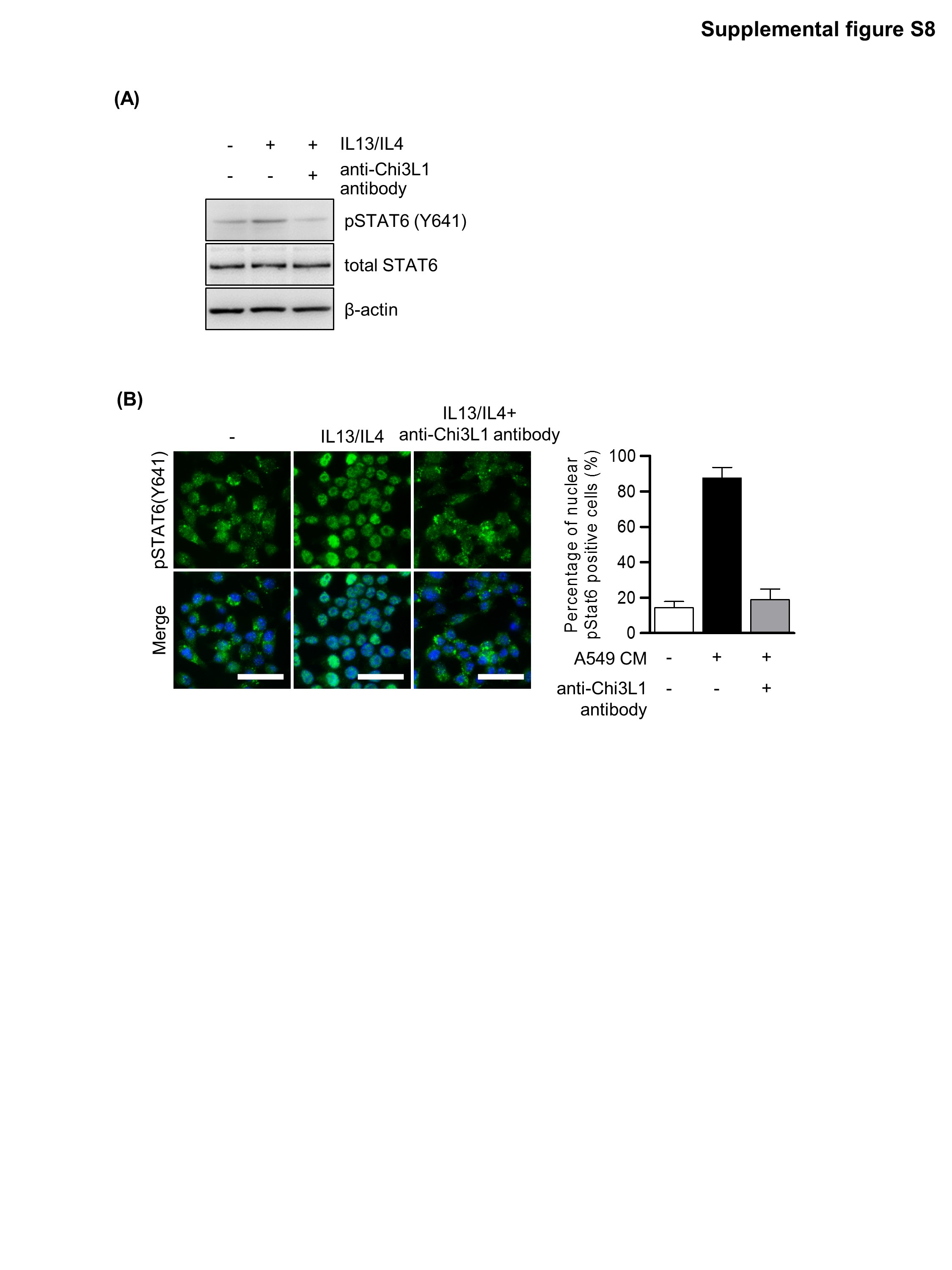


**Supplemental figure S8. STAT6 is involved in the anti-Chi3L1 antibody-induced inhibition of M2-like macrophages polarization in RAW 264.7 cells.**

(A-B) RAW 264.7 macrophages were stimulated with LPS (100 ng/ml) with or without anti-Chi3L1 antibody (1 μg/ml). (A) Western blot was performed to measure the p-STAT6. (B) The fixed cells were immunofluorescence stained with p-STAT6. The percentage of nuclear localized p-STAT6 cells was calculated. Data are presented as mean ±SD from three independent experiments. Scale bar, 50 μm.


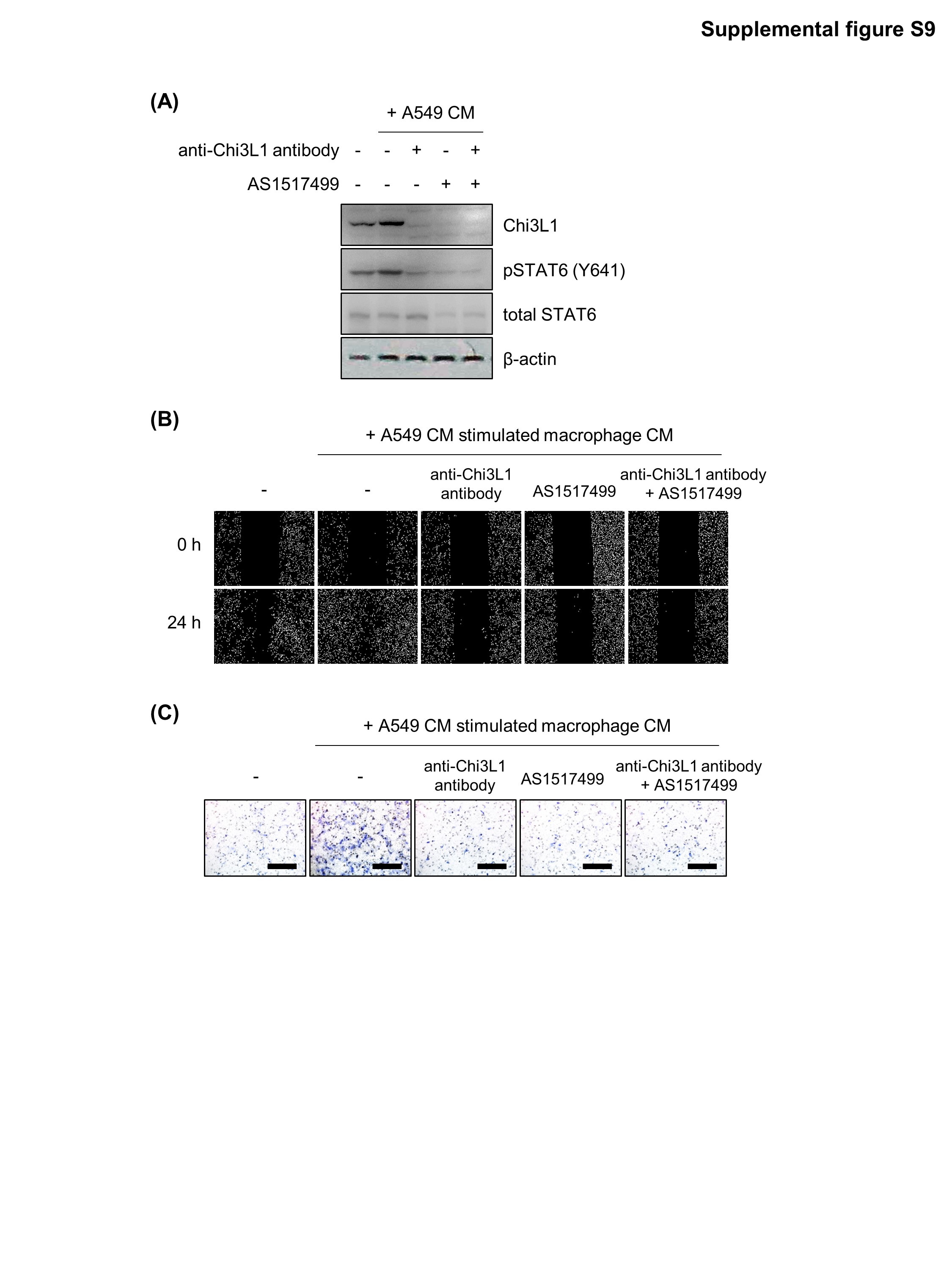


**Supplemental figure S9. The combination anti-Chi3L1 antibody and AS1517499 treatment has no additive effect on tumor migration.**

(A) Thp-1 cells were treated with PMA (100 ng/ml) and stimulated with A549 CM with or without anti-Chi3L1 antibody (1 μg/ml) or pSTAT6 inhibitor AS1517499 (100 nM). Western blot was performed to measure the p-STAT6. (B-C) Thp-1 cells were treated with PMA (100 ng/ml) and stimulated with A549 CM with or without anti-Chi3L1 antibody (1 μg/ml) or pSTAT6 inhibitor AS1517499 (100 nM) for 24 h. The culture medium was replaced with fresh media, and the supernatant medium was collected as macrophage-CM. (B) For the wound healing assay, A549 cells were seeded in a plate, then the cells were wounded with a straight scratch using a pipette tip and treated with macrophage-CM for 18 h. (C) The effect of macrophage-CM on A549 cell migration was evaluated by a Trans-well assay. The A549 cells were seeded in the upper chamber and macrophage-CM was placed into the lower chambers and incubated at 37 °C for 18 h. The migrated cells on the bottom chamber were stained with 0.1% crystal violet. Scale bar, 100 μm.


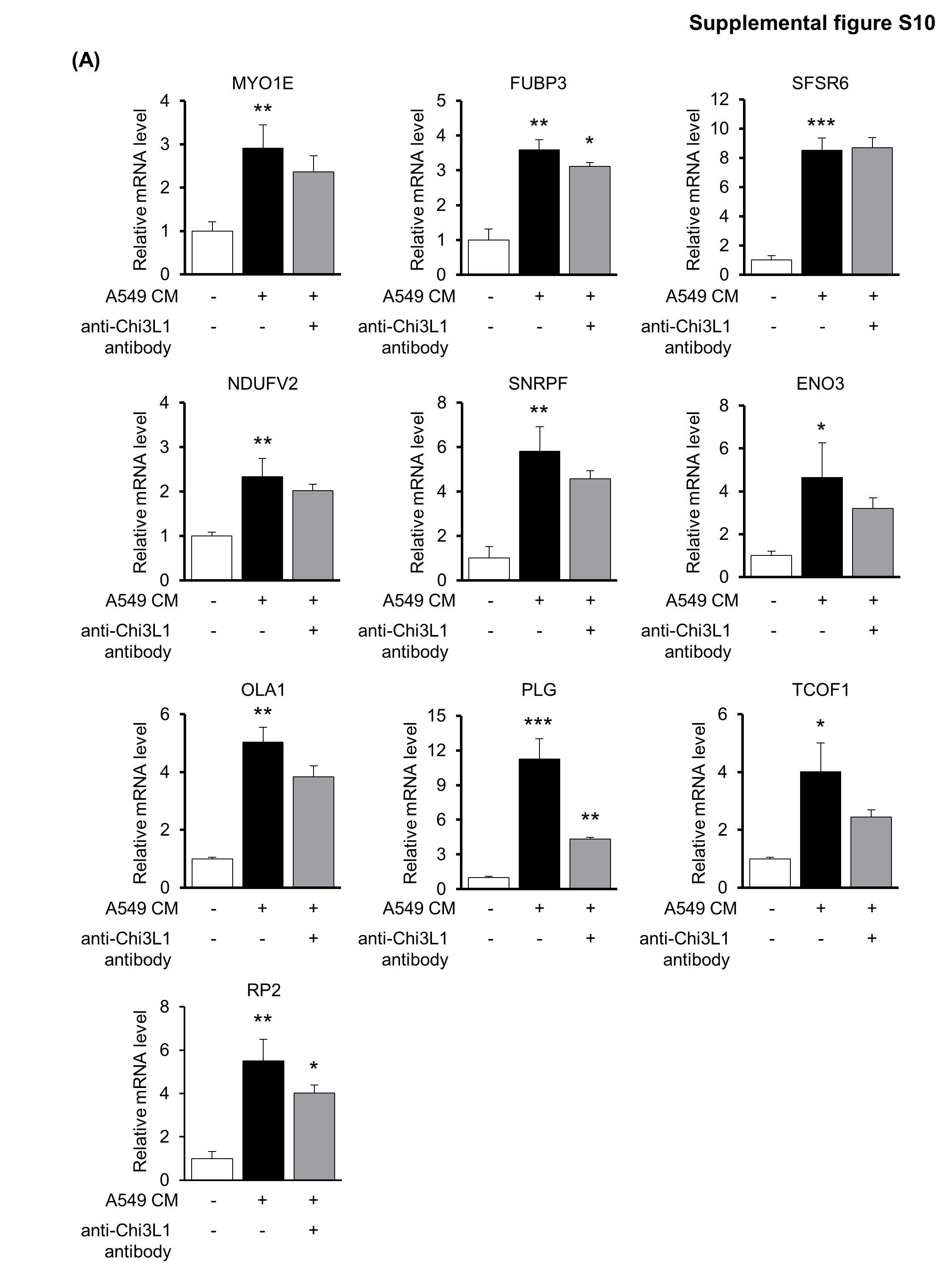

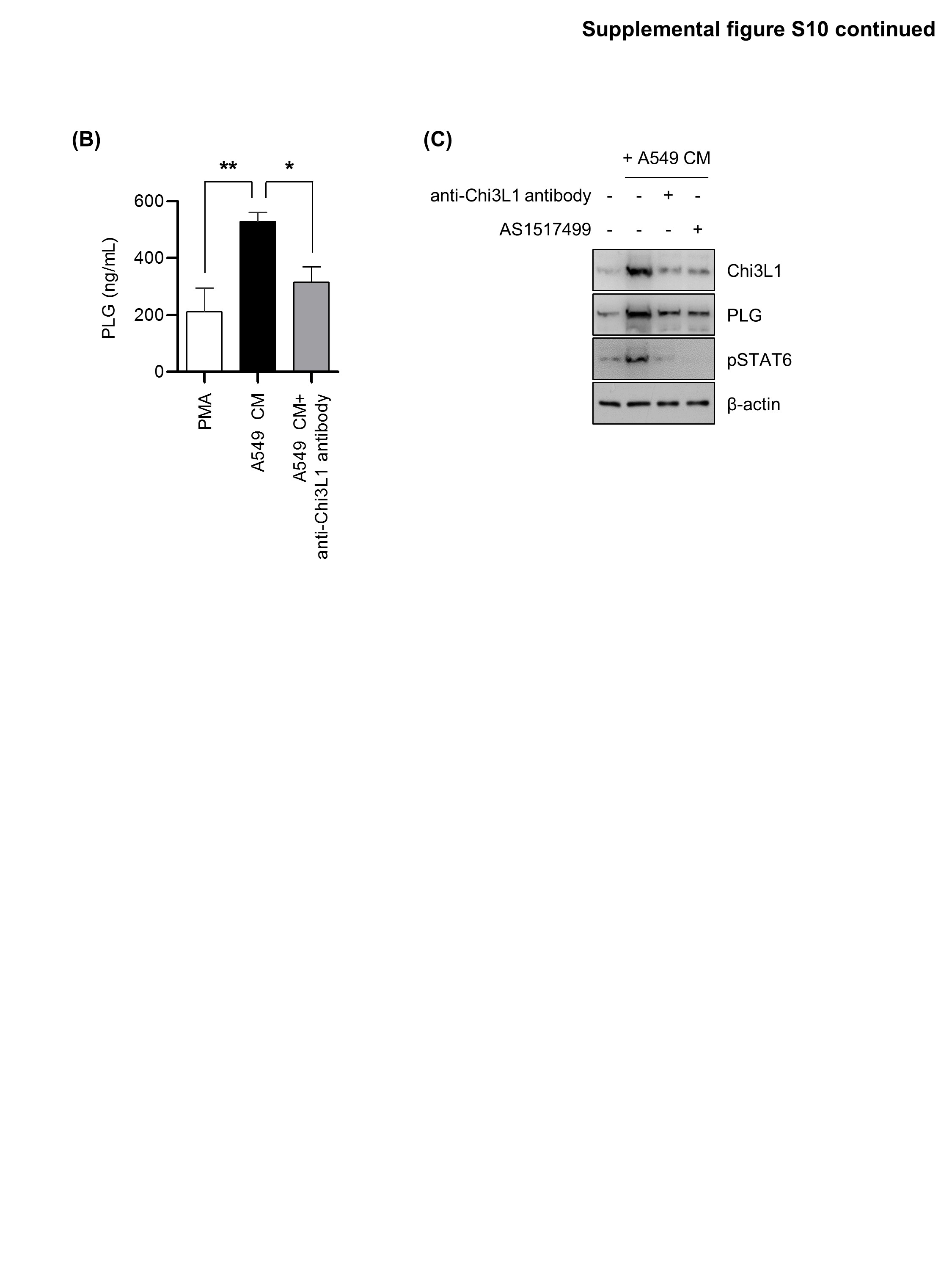


**Supplemental figure S10. The mRNA expression of putative Chi3L1 target genes and expression of PLG *in vivo* and *in vitro.***

(A) Thp-1 cells were treated with PMA (100 ng/ml) and stimulated with A549 CM with or without anti-Chi3L1 antibody (1 μg/ml). RT-qPCR analysis of indicated gene mRNA levels. Data are presented as mean ±SD from three independent experiments. *, P<0.05; **, P<0.01; ***, P<0.001. (B) Thp-1 cells were treated with PMA (100 ng/ml) and stimulated with A549 CM with or without anti-Chi3L1 antibody (1 μg/ml). Quantification of PLG by ELISA. Data are presented as mean ±SD from three independent experiments. *, P<0.05; **, P<0.01. (C) Thp-1 cells were treated with PMA (100 ng/ml) and stimulated with A549 CM with or without anti-Chi3L1 antibody (1 μg/ml) or pSTAT6 inhibitor AS1517499 (100 nM). Western blot was performed to measure the PLG protein level.
